# Effects of gut barrier dysfunction during a viral respiratory disease challenge on immune function of feedlot beef calves

**DOI:** 10.1093/jas/skag117

**Published:** 2026-04-10

**Authors:** Ryan C Foster, Vinicius N Gouvêa, Matthew R Beck, Oscar J Benitez, Josue Diaz-Delgado, Kagan F Migl, Fernanda Rosa, Matthew A Scott, Nathan S Long, John T Pennington, Reinaldo F Cooke

**Affiliations:** Texas A&M AgriLife Research, High Plains Research and Extension Center, Canyon, TX, 79015, United States; Department of Animal Science, Texas A&M University, College Station, TX, 77845, United States; Texas A&M AgriLife Research, High Plains Research and Extension Center, Canyon, TX, 79015, United States; Department of Animal Science, Texas A&M University, College Station, TX, 77845, United States; Department of Animal Science, Texas A&M University, College Station, TX, 77845, United States; Department of Veterinary Sciences, Texas Tech University, Lubbock, TX, 79409, United States; Texas A&M Veterinary Medical Diagnostic Laboratory, College Station, TX, 77843, United States; School of Veterinary Medicine, Texas Tech University, Amarillo, TX, 79106, United States; School of Veterinary Medicine, Texas Tech University, Amarillo, TX, 79106, United States; College of Veterinary Medicine and Biomedical Sciences, Texas A&M University, Canyon, TX, 79015, United States; Department of Animal Science, Texas A&M University, College Station, TX, 77845, United States; Texas A&M AgriLife Research, High Plains Research and Extension Center, Canyon, TX, 79015, United States; Department of Animal Science, Texas A&M University, College Station, TX, 77845, United States

**Keywords:** bovine respiratory disease, inflammation, immune response, leaky gut, stress

## Abstract

Feedlot morbidity and mortality have increased in recent decades, driven in part by the prevalence of bovine respiratory disease (BRD). Research on gut barrier dysfunction (GBD) reveals similarities in predisposing factors and etiopathogenic mechanisms to BRD. This overlap suggests that GBD may serve as a predisposing factor, increasing susceptibility to BRD. To explore this connection, 15 Angus × Holstein heifers (initial body weight = 475 ± 12 kg) were used in a randomized complete block design experiment to evaluate the effects of induced changes in intestinal permeability on immune responsiveness during a viral respiratory disease challenge. Treatments were control (CT; *n* = 7) or GBD (*n* = 8), where GBD heifers underwent a protocol to increase gut permeability using aspirin (100 mg/kg of body weight every 12 h for 4 consecutive days). Daily dry matter intake and average daily vaginal temperature (DVT) were continuously recorded. After aspirin withdrawal, all heifers were inoculated with bovine herpesvirus-1. Complete blood count, cytokines, acute-phase proteins (APP), cortisol, and intestinal morphology were evaluated. Heifer was considered the experimental unit for all analyses. The statistical model included the fixed effect of treatment and hour/day and the resultant interactions, run and heifer (treatment) were used as random effects, and hour or day was the term for all repeated statements, and heifer (treatment) was the subject. Aspirin administration increased gut permeability in GBD heifers, as evidenced by greater plasma Chromium-EDTA recovery (*P *= 0.02) and increased lipopolysaccharide-binding protein concentration (treatment × hour; *P *< 0.01) early in the disease challenge. Compared to CT, GBD heifers tended to exhibit decreased DVT (*P *= 0.10) and haptoglobin concentration (treatment × hour; *P *= 0.06) by the end of the disease challenge. No significant differences were observed in serum amyloid A, interleukin-6, tumor necrosis factor-α, interleukin-10, or cortisol concentration (*P *≥ 0.20). A tendency for decreased white blood cell (*P *= 0.08) and lymphocyte counts (treatment × hour; *P *= 0.08) in GBD heifers was observed. There were no effects of treatments on intestinal morphology (*P *≥ 0.16). These findings suggest that increased gut permeability influences immune responses by reducing the febrile response and decreasing the production of some APP. Greater emphasis on gut health could improve disease outcomes in BRD management.

## Teaser

Heifers with increased gut permeability during a respiratory disease challenge showed a decreased febrile response and altered acute-phase protein production. These findings suggest potential impacts on immune function and disease resolution, highlighting the importance of gut health in promoting animal health.

## Introduction

Cattle are often subjected to a variety of stressors in production environments and along the market chain (Carroll and Forsberg 2007; Cooke 2017; Galyean et al. 2022), and for decades researchers have observed that these stress events can suppress immune function and increase the risk of disease (Roth and Kaeberle 1982; Blecha et al. 1984; Roth 1985; Duff and Galyean 2007; Cooke 2017). Among the greatest consequences of altered immune functions is the bovine respiratory disease (BRD) complex, which has a considerable economic impact on the beef cattle industry (Galyean et al. 2022). BRD is an economically costly disease as it is the leading cause of morbidity and mortality in feedlot cattle production (Woolums et al. 2005; Galyean et al. 2022). Estimates of expenses and lost profits due to BRD have been reported to be as much as $2 billion annually (Powell 2013; Wilson et al. 2017). According to an industry survey, BRD accounts for approximately 75% of feedlot morbidity and greater than 50% of feedlot mortality (Edwards 1996). Thereby, it has previously been estimated that morbidity associated with BRD among all feedlot cattle in the United States is approximately 16.2% (Nahms 2013). Cattle affected with BRD exhibit negative impacts on feedlot performance, including lower ADG early in the finishing phase that results in longer days on feed, reduced final weights, and decreased carcass weight and marbling scores (Gardner et al. 1999; Holland et al. 2010; Beck et al. 2025). Gardner et al. (1999) reported that steers treated for BRD exhibited a decrease in ADG of 4%, a decrease in final BW of 1.7%, and a decrease in hot carcass weight of 2.6% compared to non-treated healthy steers. Furthermore, increasing feedlot death loss has been observed in recent decades (Loneragan et al. 2001; Babcock et al. 2006; Vogel et al. 2015), which is attributable in part to BRD (Vogel et al. 2015). These pernicious trends necessitate seeking new strategies and mitigation measures to reduce the impact of respiratory disease on cattle production.

Recent studies have emphasized the link between stress in livestock and gut barrier dysfunction (GBD) (Moeser et al. 2007a; Zhang et al. 2013; Pohl et al. 2017; Pisoni et al. 2022), a condition that increases gut permeability and allows immunogenic bacteria to enter circulation, triggering inflammation and immune activation (Lambert 2009). Due to their shared stress-related etiologies, GBD likely coincides with BRD infection in stressed cattle. An inflammatory response associated with GBD may divert resources away from other immune functions, potentially compromising the ability to combat BRD. Exploring the interplay between gut and respiratory health could help to elucidate mechanisms behind the significant economic losses associated with stress in cattle production.

Immune activation from stress and disease carries a high metabolic cost; for example, an acutely activated immune system in Holstein cows uses over 1 kg of glucose in a 12-h period (Kvidera et al. 2017b). More specifically, inflammation from increased endotoxin load, such as in the case of GBD, is found to lead to negative nitrogen balance and a 30% increase in energy expenditure (Lochmiller and Deerenberg 2000; Mani et al. 2012). Given that the gut is the body’s largest immune organ by surface area (Mani et al. 2012; Stewart et al. 2017), and that 70%–80% of immune cells reside in the gut (Wiertsema et al. 2021), GBD could impose a significant strain on immune and energy resources. The impacts of immune activation on reducing feed intake, growth rates, and feed efficiency are further compounded under the dual burden of a simultaneous BRD and GBD disease state (Hanssen et al. 2004; Mani et al. 2012).

We hypothesize that GBD-induced inflammation creates an increased strain on resources that compromises immune function during BRD infection, creating a compounding negative effect on health and performance. This study aimed to assess how increased gut permeability impacts the immune response in cattle challenged with respiratory disease through hematologic, immunologic, serologic, endocrinologic, molecular, and histopathologic analyses.

## Materials and methods

The study was conducted at the joint Texas A&M AgriLife Research/USDA-ARS Research Feedlot in Bushland, TX. All procedures involving live animals were reviewed and approved by the West Texas A&M University Institutional Animal Care and Use Committee (protocol number 2023.01.003). The research protocol was also approved by the Institutional Biosafety Committee of Texas A&M University (protocol number IBC2023-013).

### Animals, feeding, and housing

**Figure 1:**
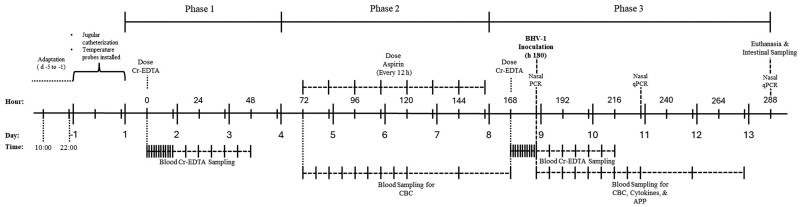
Timeline of major events throughout the experiment. Phase 1 (covariate period), from days 1 to 3, was used to determine baseline measurements for each heifer of Cr-EDTA recovery, dry matter intake, and vaginal temperature as covariates for statistical analysis. Phase 2, from days 4 to 7, included administering aspirin via balling gun (100 mg/kg of body weight [BW]) to the gut barrier dysfunction group (GBD; *n* = 8) every 12 h from days 4 to 7 and was used to examine the effects of aspirin administration on dry matter intake, vaginal temperature, and complete blood count measurements. The control group (CT; *n* = 7) did not receive aspirin. Phase 3, from days 8 to 13, began when all heifers (*n* = 15) were inoculated with Bovine Herpesvirus-1 (BHV-1) and was used to examine the effects of intentionally induced increased gut permeability on Cr-EDTA recovery, dry matter intake, vaginal temperature, complete blood count measurements, serum cytokine concentrations, acute-phase protein concentrations, and cortisol concentration. APP, acute-phase protein; CBC, complete blood count; Cr-EDTA, chromium-ethylenediaminetetraacetic acid; qPCR, quantitative polymerase chain reaction.

Fifteen Angus × Holstein crossbreed heifers were obtained from a single dairy farm (Red Rock Dairy, Amherst, TX) and transported for approximately 1 hour to the research facility where they were acclimated to the new feedlot setting and diet four months before the beginning of the experiment. Pre-trial blood samples were collected on day 5 (Figure 1) via jugular venipuncture using a 20 Ga × 1 ½” needle (Greiner Bio-One North America Inc., Monroe, NC) into 10 mL tubes containing clot activator (BD, Franklin Lakes, NJ) for determination of serum antibody titers for bovine herpesvirus-1 (BHV-1), measured via virus neutralization by the Texas A&M Veterinary Medical Diagnostic Laboratory (Canyon, TX) to assure all heifers were negative for BHV-1. Before the beginning of the study, heifers were allowed a 5-d acclimation to the experimental setting, which consisted of 1.0 × 2.5 m individual pens with rubber flooring that are equipped with automatic water fountains and individual feeders in a forced ventilation barn. Heifers were approximately 14 months ± 3 days of age at the beginning of the experiment. The experiment was conducted over a 13-d period (Figure 1), consisting of a 3-d covariate period (Phase 1), and 10 days of sampling (Phases 2 [4 d] and 3 [6 d]). At the beginning of the experiment (days −1 and 1), individual paired-day unshrunk body weight (BW) was collected to account for differences in gut fill (initial BW = 475 ± 12 kg), and heifers were blocked in two BW blocks. Initial BW, BHV-1 antibody titers, and sire information were used to balance heifers into the treatment groups within each BW block in a randomized complete block design. Treatments were control (CON; *n* = 7) or intentionally induced increased gut permeability (GBD; *n* = 8) by dosing aspirin (100 mg/kg of BW PO every 12 h) as described by Briggs et al. (2020) for 4 d.

All heifers were fed a basal diet (Table 1) formulated to meet the nutrient requirements of growing cattle (National Academies of Sciences, Engineering, and Medicine (NASEM) 2016) during the entire experiment, which was delivered as a total mixed ration. Heifers were fed once daily, starting at 0800 h. Individual feeders were managed for *ad libitum* intake (approximately 2% orts). Refusals were removed daily, weighed, and sampled for dry matter determination and dry matter intake (DMI) calculation. Diet samples were collected daily and composited by week (*n* = 2) for analysis of nutrient composition.

**Table 1:** Ingredient and chemical composition of the basal diet (dry matter [DM] basis).

Ingredients, %	
**Wheat hay**	50.0
**Steam-flaked corn**	20.0
**Dry Distillers’ grains with solubles**	5.00
**Wet corn gluten feed^1^**	20.0
**Mineral and vitamin premix^2^**	5.00
**Analyzed chemical composition, %**	
**Dry matter, as fed**	66.9
**Crude Protein**	15.9
**Ether extract**	3.20
**Neutral detergent fiber**	41.0
**Acid detergent fiber**	21.1
**Lignin**	4.40
**Ash**	10.7
**Net energy of maintenance, Mcal/kg^3^**	1.59
**Net energy of gain, Mcal, kg^3^**	0.970

1 Cargill, Sweet Bran, Dalhart, TX.

2 Containing (DM basis): 338.3 g/kg Calcium Carbonate, 337.8 g/kg Dried Distillers Grains (as a carrier), 204.5 g/kg Magnesium Sulfate, 69.5 g/kg Salt, 39.9 g/kg Potassium Chloride, 2.5 g/kg Manganese Sulfate, 1.4 g/kg Zinc Oxide, 830 mg/kg Copper Sulfate, 415 mg/kg Sodium Selenite, 16.1 mg/kg Cobalt Carbonate, 12.8 mg/kg EDDI, 56,587 IU/kg Vitamin A, 5622 IU/kg Vitamin D, and 706 IU/kg Vitamin E. Monensin was included to provide 33 mg/kg of the total mixed ration.

3 Expected values were calculated using tabular values from NASEM (2016).

### Treatments and sampling

On day −1, all heifers were fitted with indwelling vaginal temperature recording devices seated within non-hormonally active controlled intravaginal drug release (CIDR) devices (Pfizer Animal Health, Sterling, CO) to measure approximate core body temperature as described in Burdick et al. (2012). Briefly, the hormone coating was removed, and the frame was washed to remove any remaining progesterone residue. The existing notches were drilled out to fit Star-Oddi DST micro-T temperature probes (MeterMall USA, Marysville, OH) protected by a thin covering of aluminum foil. Heat shrink tubing was used to maintain the probes in their position and the entire CIDR assembly was coated in a multi-purpose liquid rubber coating (Flex Seal, Weston, FL) to affix the modifications and reduce abrasiveness in the vaginal canal. Vaginal temperature was recorded within individual heifers at 1-min interval throughout the entire experiment and was averaged daily by treatment to report average daily vaginal temperatures (DVT).

Also on day 1, a licensed veterinarian placed jugular catheters for central blood collection using 14Ga × 13 cm extended-use intravenous catheters (MILA International, Inc., Florence, KY) according to Sharon et al. (2019). A 1 cm-long incision was made in the skin to allow the passage of the catheter into the jugular vein. The catheters were fixed in position using 2-0 nylon suture (Ethicon, Inc., Raritan, NJ) at the winged attachment points, and the entire neck region was wrapped with 10 cm. Elastikon bandaging (Johnson and Johnson, New Brunswick, NJ). A 13 Ga × 76 cm tubing extension (MILA International, Inc., Florence, KY) was used to allow for blood collection with minimal cattle handling. At each blood collection, approximately 2 mL of sterile saline was pushed through the catheter tubing to free obstructions, and the first 10 mL of blood and saline were collected into a waste syringe and discarded. The blood sample was collected into a sterile syringe and transferred into their respective BD vacutainer tubes for each time point as follows: serum samples were collected into 8.5 mL serum separator tubes, plasma samples were collected into 10 mL sodium heparin tubes, whole blood was collected into 3 mL K_2_ EDTA tubes, and plasma samples for Cr-EDTA analysis were collected into 6 mL trace element K_2_ EDTA tubes (BD, Franklin Lakes, NJ). After each sample collection, 15 mL of heparinized saline was used to flush the catheter and prevent clotting within the catheter or extensions. The catheter extension tubing was clamped between blood sample collections, and the tubing was held in place within the bandaging.

Aspirin (i.e. acetylsalicylic acid; AHC Products Inc., Winchester, KY) was dosed orally within 24 mL porcine hard gelatin capsules (Torpac Inc., Fairfield, NJ) using a balling gun at a dosage of 100 mg/kg every 12 h during four consecutive days (day 4 to 7, Phase 2; Figure 1). After a 24 h withdrawal from the last dose of aspirin, as recommended by Smith et al. (2008) for meat and milk withdrawal, heifers were inoculated on day 8 with 1.5 × 10^8^ pfu of bovine herpesvirus-1 as described by Sharon et al. (2019) and (Broadway 2021). The BHV-1 inoculum used in this study was the Cooper strain (Texas Vet Lab Inc., San Angelo, TX). Briefly, individual vials of BHV-1 were reconstituted with 2 mL of sterile 1× phosphate-buffered saline. Two syringes per calf, each containing 1 mL of the reconstituted virus, were fitted with a mucosal atomization device (Teleflex Medical, Wayne, PA), and each heifer was inoculated with 1 mL of the BHV-1 containing solution per nostril (Sharon et al. 2019).

### Gut permeability

Total gut permeability was evaluated by measuring the recovered amount of plasma concentrations of chromium-ethylenediaminetetraacetic acid (Cr-EDTA), an indigestible marker used to measure epithelial paracellular permeability, as described by Horst et al. (2020). The Cr-EDTA solution was prepared according to Binnerts et al. (1968) using a protocol developed by Horst et al. (2020) to obtain a 179-mM solution. In brief, using a 500 mL beaker, 47.7 g of chromium (III) trichloride hexahydrate was dissolved into 300 mL of distilled water. In a separate 1 L beaker, 66.7 g of Na_2_-EDTA dihydrate was added and dissolved into 500 mL of distilled water using heat and a magnetic stir bar for 2 hours. The solutions were combined into a new beaker, covered with aluminum foil, and gently boiled for 1 hour (at 110°C). The resulting solution changed color to purple and was allowed to cool and stir overnight. Then, 1.937 g of CaCl_2_ solution was added to precipitate any unreacted EDTA, which dissolved as the pH decreased. The final solution was brought to a pH of 6.0 using NaOH pellets.

The procedure to measure total gut permeability based on plasma recovery of Cr-EDTA was implemented as follows: all heifers received 0.1 g/kg of BW of the 179-mM Cr-EDTA solution using an esophageal tube and Frick’s speculum, followed by 1 L of water, and blood samples were collected at hours −0.5, 0, 1, 2, 3, 4, 5, 6, 7, 8, 9, 10, 11, 12, 18, 24, 30, 36, 42, and 48 relative to dosing of Cr-EDTA (Figure 1). This procedure occurred twice: once before aspirin administration from day 1 to 3 to measure baseline permeability (Phase 2), and a second time 12 hours after the final dose of aspirin from day 8 to 10, to examine the effect of the treatment (Phase 3). Following the first Cr-EDTA administration and sampling period, a 24-hour washout period from days 3 to 4 was implemented to allow for clearance of any remaining marker. Blood samples were centrifuged at 2,500 × *g* for 30 min and plasma aliquots were frozen for Cr-EDTA analysis using plasma mass spectrometry (Michigan State Veterinary Diagnostic Laboratory; Lansing, MI). Baseline plasma Cr-EDTA recovery for each heifer, before aspirin administration from days 1 to 3, was used as a covariate for Cr-EDTA recovery from days 8 to 10, after the treatment was applied. Total Cr-EDTA recovery was determined using the area under the curve of plasma Cr-EDTA concentration across the time points, which was calculated as described by Foote et al. (2024) using GraphPad Prism 10 (GraphPad Software, San Diego, CA).

### Immune response

Blood samples were collected to measure the hematological and immunological responses to aspirin administration in Phase 2 and the BHV-1 challenge in Phase 3. Whole blood was collected from days 4 to 8 at 0, 6, 12, 18, 24, 30, 36, 42, 48, 72, and 96 h from the first dose of aspirin in Phase 2 to examine its effects on complete blood count (CBC) measurements (Figure 1). Whole blood samples were analyzed for CBC using a Vetscan HM5 automated hemocytometer (Zoetis Animal Health, Parsippany, NJ). The resulting 23-parameter CBC included measurements of white blood cell count (WBC) lymphocyte count (LYM), lymphocytes as a percentage of WBC (LYM%), monocyte count (MON), monocytes as a percentage of WBC (MON%), neutrophil count (NEU), neutrophils as a percentage of WBC (NEU%), basophil count (BAS), basophils as a percentage of WBC (BAS %), eosinophil count (EOS), eosinophils as a percentage of WBC (EOS%), red blood cell count (RBC), hematocrit (HCT%), red cell distribution width (RDW), red cell distribution width ratio (RDW%), mean corpuscular hemoglobin (MCH), mean corpuscular hemoglobin concentration (MCHC), mean corpuscular volume (MCV), platelet count (PLT), plateletcrit (PCT%), platelet distribution width (PDW), platelet distribution width ratio (PDW%), and mean platelet volume (MPV). Blood serum, plasma, and whole blood were collected on days 8 to 12 at 0, 6, 12, 18, 24, 30, 36, 42, 48, 72, and 96 h relative to BHV-1 inoculation in Phase 3 to examine the effect of increased gut permeability on CBC measurements, serum cytokines (interleukin-6 [IL-6], tumor necrosis factor-α [TNF-α], and interleukin-10 [IL-10]), acute-phase proteins (lipopolysaccharide binding protein [LBP], haptoglobin, and serum amyloid A [SAA]), and the glucocorticoid response (cortisol; Figure 1). After blood collection, serum and plasma samples were obtained through centrifugation at 2,500×g for 30 min at 4°C and aliquots were frozen (−20°C). The concentrations of IL-6 (kit #ELB-IL6-1; RayBiotech, Inc., Norcross, GA), TNF-α (kit #ELB-TNFa-1; RayBiotech, Inc.), IL-10 (kit #ELB-IL10-1; RayBiotech, Inc.), LBP (kit #HK503; Hycult Biotech, Uden, Netherlands), and SAA (kit #TP-802; Tridelta Development Ltd., Kildare, Ireland) in serum or plasma were determined using bovine-specific commercial sandwich enzyme-linked immunosorbent assay (ELISA) kits. Serum dilutions used for analysis of IL-6, TNF-α, IL-10 were 1:2. Serum dilutions for LBP and SAA were 1:1,000 and 1:2,000, respectively. Colorimetric absorbance was read using an optical density of 450 nm in a Synergy H1 spectrophotometer (BioTek, Charlotte, VT). The ELISA assays conducted in our lab had intraassay CV for IL-10, TNF-α, IL-6, LBP, and SAA of 1.71, 4.81, 14.92, 4.40, and 3.77%, respectively. The interassay CV for IL-10, TNF-α, IL-6, LBP, and SAA assays were 19.10, 142.43, 51.67, 21.42, and 19.25, respectively. The high interassay CVs among the TNF-α and IL-6 assays were due to the extremely low concentrations of the analyte in the interassay control samples that were used, which occasionally resulted in undetectable values. Haptoglobin concentrations were determined using the colorimetric procedures described by Cooke and Arthington (2013). Plasma concentrations of cortisol were determined by radioimmunoassay (No. 07221106, MP Biomedicals, Santa Ana, CA) according to Burdick et al. (2009). Interassay and intraassay CV for haptoglobin and cortisol assays was <10%.

Nasal swabs were collected immediately before the BHV-1 challenge, on day 8, and after, on day 10 and 13. A superficial nasal swabbing procedure was employed, according to Pickett et al. (2023), using a 20-cm DNA-free swab (Puritan Medical Products Company LLC, Guilford, Maine) that was aseptically introduced approximately 15 cm into each nostril and rotated around the sides of the nasal passage. The swabs were then replaced into their sterile containers along with 2 mL of sterile saline, flash-frozen with liquid nitrogen, and stored at −80°C. At the end of the experiment, the nasal swab samples were submitted to Texas A&M Veterinary Medical Diagnostic Laboratory (Canyon, TX) for quantitative polymerase chain reaction (qPCR) analysis of BHV-1 viral load as described in Broadway et al. (2021).

### Intestinal sampling and histology

On day 13, all heifers were euthanized by a licensed veterinarian with a CASH Special captive bolt gun (Accles and Shelvoke Ltd., Sutton Coldfield, West Midlands, UK), and intestinal tissues (duodenum, jejunum, and ileum) were collected immediately following euthanasia for histological analysis as described by Opgenorth et al. (2021). Intestinal samples were ∼20–30 cm in length and collected as follows: duodenum samples were collected approximately 15 cm distal to the pyloric sphincter, jejunum samples were collected approximately 80 cm distal to the pyloric sphincter, and ileal samples were collected approximately 15 cm proximal to the ileocecal junction. The samples obtained were flushed with cold phosphate-buffered saline to wash away feed contents and fixed in 10% neutral buffered formalin at a 10:1 volume ratio. Samples were used for morphometric measurements of intestinal villus height, villus width, and crypt depth as described by Horst et al. (2020).

Formalin-fixed tissues were processed and digitalized at the Texas Veterinary Medical Diagnostic Laboratory (College Station, Texas). In brief, approximately 4 mm subsamples from intestinal tissues were obtained and placed onto a slotted cassette (Epredia, Kalamazoo, MI) for tissue processing using an Excelsior AS (Epredia, Kalamazoo, MI) to remove water and embed tissues with paraffin wax (Leica Biosystems Richmond, Inc., Richmond, IL). Processed tissues were then embedded into paraffin wax blocks in tissue embedding molds using a HistoStar Embedding Workstation (Thermo Scientific, Waltham, MA). The tissue blocks were then sliced to a thickness of 4 µm with an HM 325 Rotary Microtome (Epredia, Kalamazoo, MI). Microtome ribbons were transferred to a slide (StatLab, McKinney, TX) using a histological bath (Cancer Diagnostics, Inc., Durham, NC). Slides were stained with hematoxylin and eosin and covered using a TissueTek Prisma Plus (Sakura Finetek, Torrance, CA) and scanned in a NanoZoomer S360 Digital Slide Scanner (Hamamatsu Photonics, Hamamatsu City, Japan).

Morphometric measurements of intestinal villi were taken using the NDP.view2 image viewing software (Hamamatsu Photonics, Hamamatsu City, Japan) with a procedure adapted from Opgenorth et al. (2021). Villus height was measured from the apical basement membrane of the epithelium at the villus tip to the level of the villus crypt interface along the midline. Villus width was measured at the midsection of the height of the villus from the apical to the basal basement membranes of the villus. Both sides of the villus crypt were measured from the visible villus crypt interface to the lamina propria to obtain an average measurement of crypt depth. Villus selection was based on the longest available villus on any half of a plicae circulares that was not deviated from the two-dimensional plane and had a fully discernible epithelium. When enough specimen was available, measurements were not within the same half of any plicae circularis. Ten measurements were taken per slide of the intestinal region, resulting in 30 total measurements per heifer.

### Intestinal sampling and gene expression analysis

Still on day 13, following the euthanasia, ileum samples were collected immediately after euthanasia, flash-frozen in liquid nitrogen, and then stored at −80°C until RNA processing. To evaluate the expression of inflammation-related genes as well as gene markers of intestinal integrity, total RNA was isolated from ileum samples using the RNeasy Plus Mini Kit (Kit# 74134, Qiagen), following the manufacturer’s instructions with some modifications. Briefly, prior to the RNA isolation, 1 mL of QIAzol^®^ lysis reagent (Qiagen) was added into the ileum samples (50 mg) and homogenized at 4°C, 3,000 RPM for 3 min using the Bead Blaster homogenizer (Benchmark Scientific, NJ, USA). After homogenization, 200 µL of acid phenol-chloroform (Cat# AM9720, Ambion^®^) was added to separate the RNA fraction from the organic phase. The RNA quantity and purity, as 260/280 ratio, were determined via spectrophotometry using Nanodrop One© (Thermo Scientific). The RNA integrity was assessed using the TapeStation^®^ (Agilent Technologies). The RNA quantity, purity, and integrity are presented in Table S1. The complementary DNA (cDNA) was synthesized by reverse transcription using 4 µg of total RNA and the commercial kit SuperScript IV™ (Cat# 18091050, Invitrogen) following the manufacturer’s instructions. The synthesized cDNA was then diluted 1:3 with DNase/RNase-free water. The qPCR reaction was performed in a QuantStudio (7 Pro) PCR System (Applied Biosystems) in MicroAmp^®^ Optical 384-well Reaction Plate (Applied Biosystems, USA) and SYBR^®^ Green PCR master mix (Cat# 4367659, Applied Biosystems) as described in Bionaz and Loor (2007). Samples were run in triplicate and the genes β-actin (ACTB) and β-2-microglobulin (B2M) were used as internal control genes (ICGs) as previously validated by others (Kadegowda et al. 2009; Rosa et al. 2018). The geometric mean of the ICGs was used to normalize the expression, while the relative mRNA expression of each gene was calculated based on a six-point relative standard curve using the following formula: EXP ((Ct–intercept)/slope)) Rosa (2021b). The primers used to measure the ICGs and target genes in this study were obtained from previously published studies (Table S2; Rosa 2021a; Kadegowda et al. 2009; Charavaryamath et al. 2011; Malmuthuge et al. 2013; Jacometo et al. 2015; Minuti et al. 2015; Liang et al. 2016).

### Statistical analysis

To examine the effects of the experimental procedures at different stages of the experiment, results were analyzed by the phase of the experiment, listed henceforth, where it was applicable. The first phase (Phase 1; covariate period) being before aspirin treatment from day 1 to 3; the second phase (Phase 2) being during aspirin administration from day 4 to 7; and the third phase (Phase 3) being from viral inoculation to euthanasia from day 8 to 13. The heifer was the experimental unit for all analyses. All continuous data were analyzed using the MIXED procedure of SAS (SAS Institute Inc.). Data were tested for normality and homogeneity of variance using the Shapiro-Wilks test, and data with non-normal distribution was log2-transformed prior to the statistical analysis. The Satterthwaite approximation method was used to determine the denominator degrees of freedom for tests of fixed effects. The statistical model included the fixed effect of treatment and hour/day and the resultant interactions, run and heifer (treatment) were used as random effects, and hour or day was the term for all repeated statements, and heifer (treatment) was the subject. When treatment × hour/day was significant, the effects of treatment within each timepoint was evaluated using sliced least‑squares means comparisons in PROC MIXED, applying the SLICE option with PDIFF to estimate pairwise differences. The covariance structure was selected based on the smallest Akaike information criterion. Due to the non-normal distribution of the data, the relative expression of all target genes was log-transformed (log2) prior to the statistical model analysis. Significance was defined as *P *≤ 0.05, and tendencies were defined as *P *> 0.05 and ≤ 0.10.

## Results

### Dry matter intake

**Figure 2:**
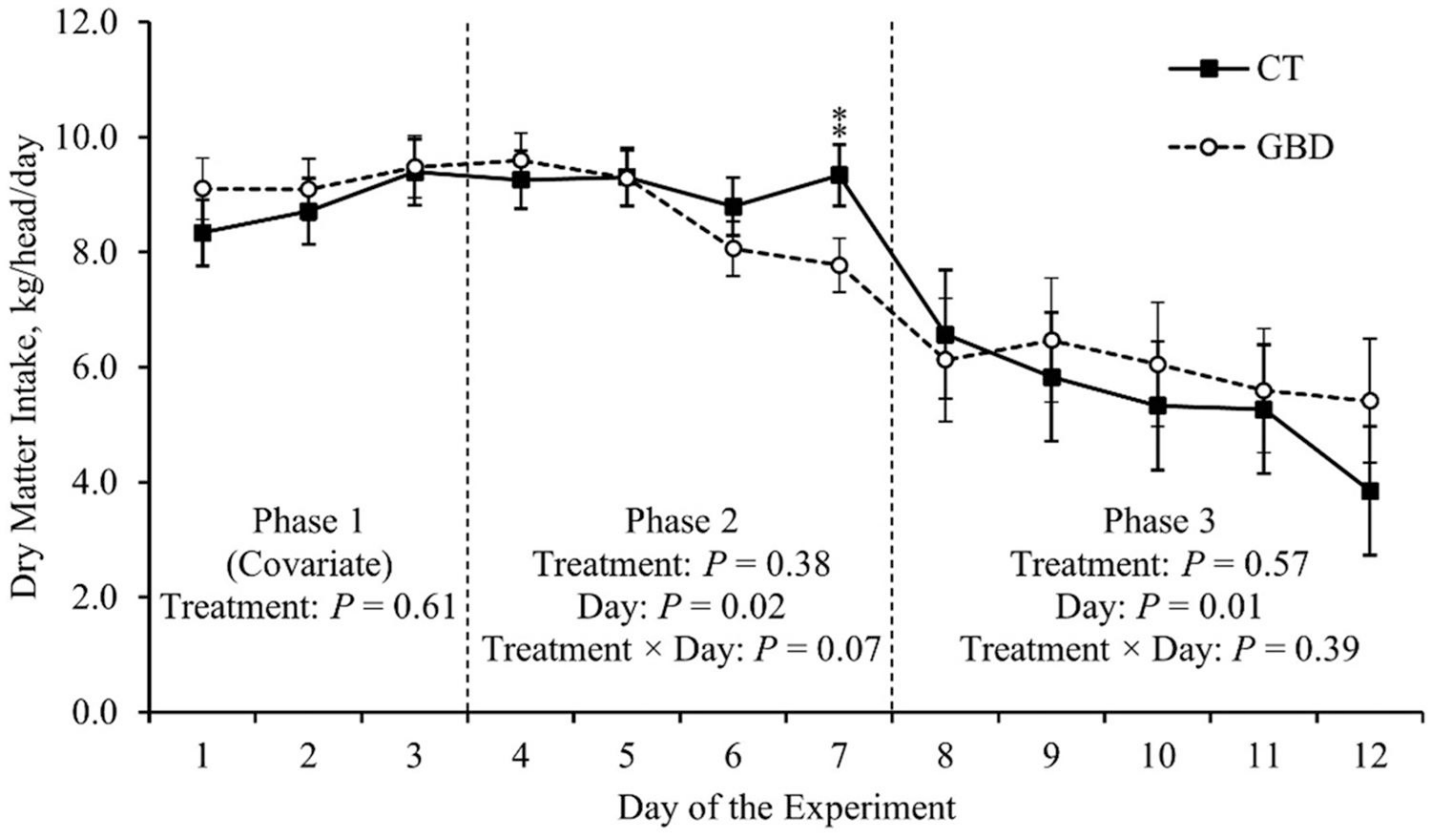
Daily dry matter intake of the basal diet throughout the experiment. Average dry matter intake in Phase 1 for each heifer was used as a covariate for statistical analysis. Phase 2 included administering aspirin via balling gun (100 mg/kg of body weight) to the gut barrier dysfunction group (GBD; *n* = 8) every 12 h from days 4 to 7 followed by a 24 h withdrawal, and the control group (CT; *n* = 7) did not receive aspirin. Phase 3 began when all heifers (*n *= 15) were inoculated with Bovine Herpesvirus-1 on day 8. ^**^P ≤ 0.05.

Daily DMI during the entire experiment is depicted in Figure 2. As designed, no differences in DMI were observed in Phase 1 between treatments (*P *= 0.61; Figure 2). There was a tendency for a treatment × day effect (*P *= 0.07) on DMI during Phase 2 (Figure 2). DMI on days 4, 5, and 6 was not statistically different between treatment groups (*P *≥ 0.30), but on day 7 of the experiment, GBD had decreased DMI (*P *= 0.04) compared to CT (Figure 2).

There was no treatment × day effect or effect of treatment in Phase 3 (*P *≥ 0.39; Figure 2). However, there was an effect of day (*P *= 0.01) on DMI in Phase 3 (Figure 2), where DMI decreased on average among both treatment groups following BHV-1 inoculation (Table 2).

**Table 2:** Effects of day on dry matter intake (DMI) and average daily vaginal temperature (DVT) of all heifers during the respiratory disease challenge (Phase 3).

Day	Item^1^	
	DMI	DVT
**8**	6.35^a^	39.53^a,b^
**9**	6.15^a^	39.12^a^
**10**	5.69^a^	39.84^b^
**11**	5.43^a,b^	39.97^b^
**12**	5.43^b^	40.07^b^
**13**	–	39.96^b^

1 Values reported are covariate (Phase 1) adjusted least square means.

a–b Values within each column with different superscripts are statistically different (*P *≤ 0.05).

### Daily vaginal temperature

**Figure 3:**
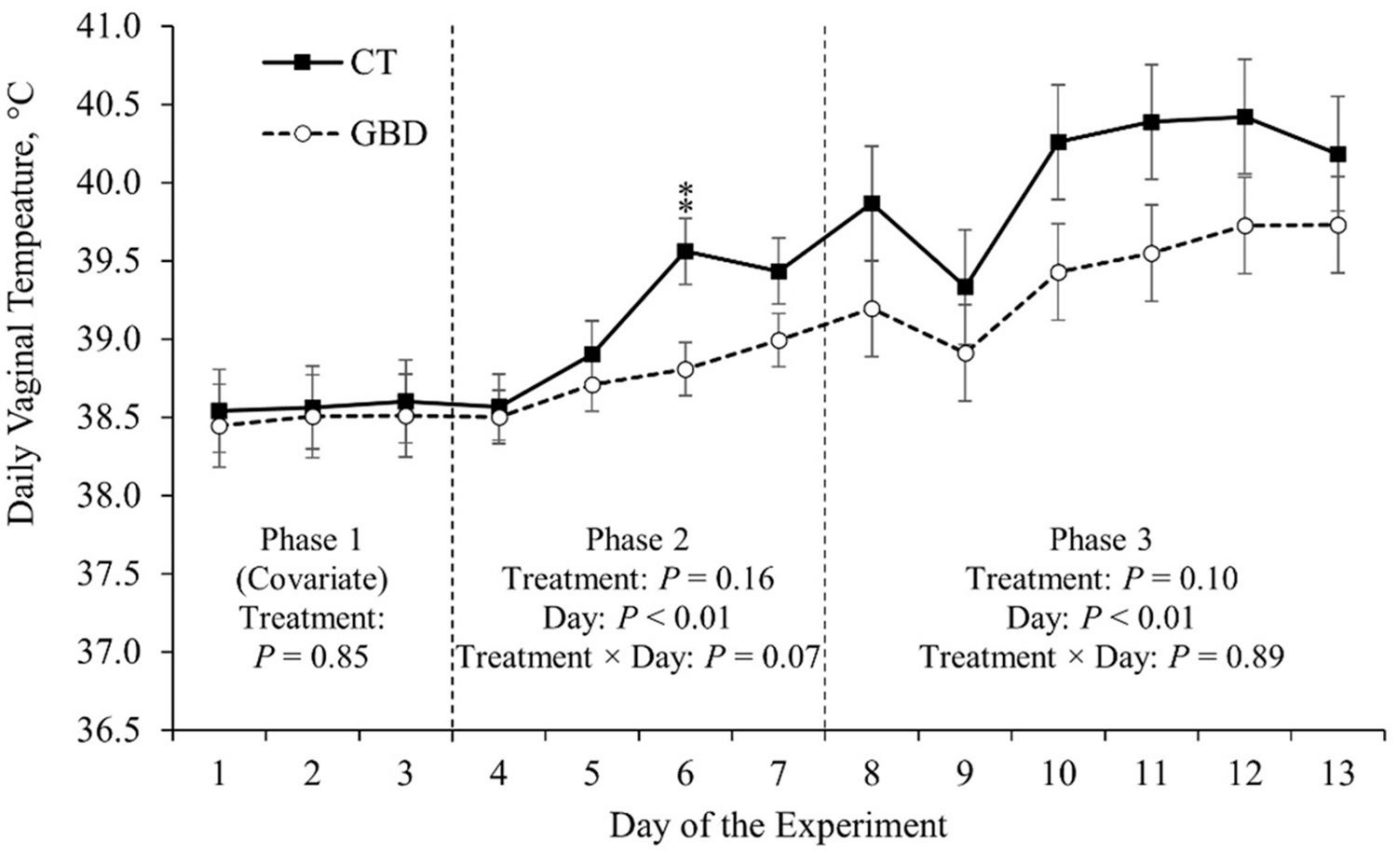
Average daily vaginal temperature throughout the experiment. Average daily vaginal temperature in Phase 1 for each heifer was used as a covariate for statistical analysis. Phase 2 included administering aspirin via balling gun (100 mg/kg of body weight) to the gut barrier dysfunction group (GBD; *n* = 8) every 12 h from days 4 to 7 followed by a 24 h withdrawal, and the control group (CT; *n* = 7) did not receive aspirin. Phase 3 began when all heifers (*n* = 15) were inoculated with Bovine Herpesvirus-1 on day 8. ^**^P ≤ 0.05.

The average DVT during the entire experiment is depicted in Figure 3. There was a tendency for a treatment × day effect (*P *= 0.07) on DVT during Phase 2 (Figure 3). DVT did not differ between CT and GBD days 4, 5, and 7 (*P *≥ 0.14), but on day 6, GBD had decreased DVT compared to CT (*P *= 0.02; Figure 3).

There was no treatment × day effect (*P *= 0.89) on DVT in Phase 3 (Figure 3). However, there was an effect of day (*P *< 0.01) on DVT in Phase 3 (Figure 3), where DVT increased in the days following BHV-1 inoculation on average among both treatment groups (Table 2). Additionally, there was a tendency for an effect of treatment (*P = *0.10) on DVT in Phase 3, where GBD had decreased mean DVT compared to CT in the days following BHV-1 inoculation (Figure 3).

### Cr-EDTA recovery

**Figure 4:**
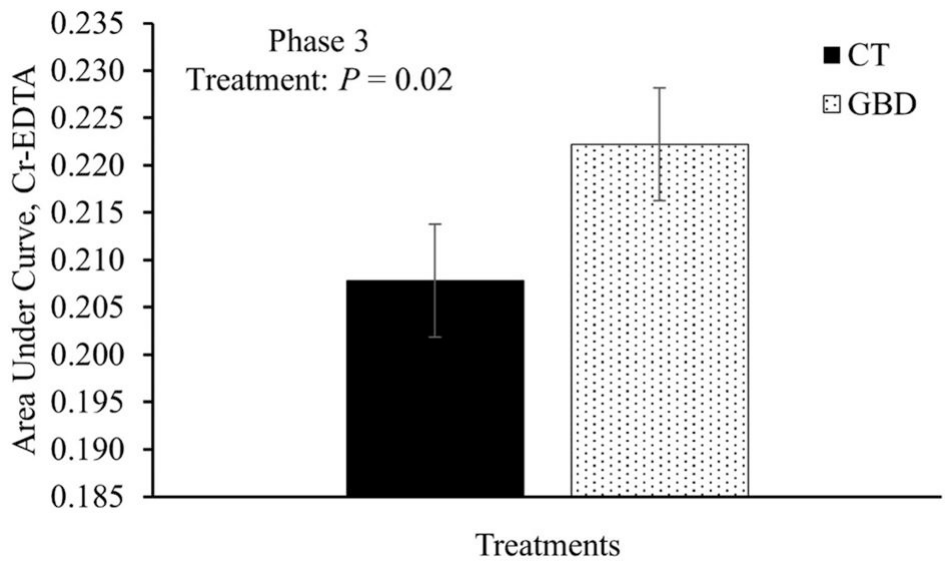
Area under the curve of plasma Cr-EDTA concentration measured from days 8 to 10. Baseline chromium concentrations for each heifer, measured prior to treatment administration from days 1 to 3 (Phase 2), were used as a covariate for statistical analysis. Aspirin was administered via balling gun (100 mg/kg of body weight) to the gut barrier dysfunction group (GBD; *n* = 8) every 12 h from days 4 to 7, and the control group (CT; *n* = 7) did not receive aspirin. All heifers (*n* = 15) received 0.1 g/kg of the 179-mM Cr-EDTA solution using an esophageal tube and Frick’s speculum, followed by 1 L of water.

Total recovery of Cr-EDTA was compared by area under the curve of plasma concentrations and is reported in Figure 4. The maximum observed Plasma Cr-EDTA concentration for all heifers was 0.009 μg/mL ± 0.00087. There was an effect of treatment on total Cr-EDTA recovery, where GBD had greater (*P *= 0.02) area under the curve of plasma Cr-EDTA concentration compared to CT (Figure 4).

### CBC

**Figure 5:**
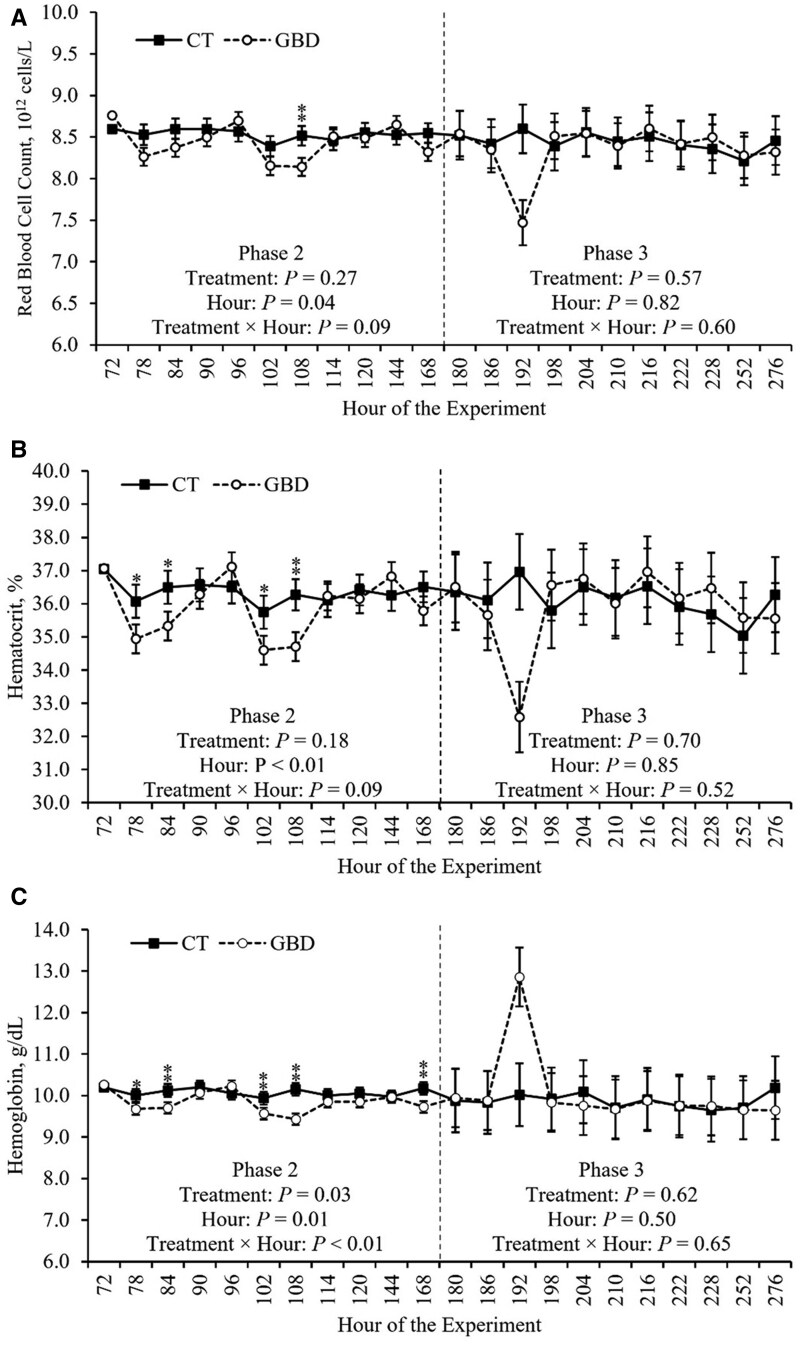
Treatment × hour effects on red blood cell and hemoglobin measurements obtained by complete blood count during Phase 2. The values reported are covariate adjusted least square means. The baseline measurement immediately prior to treatment administration, hour 72, was used as a covariate for statistical analysis. Phase 2 included administering aspirin via balling gun (100 mg/kg of BW) to the gut barrier dysfunction group (GBD; *n* = 8) every 12 h from days 4 to 7 followed by a 24 h withdrawal, and the control group (CT; *n* = 7) did not receive aspirin. Phase 3 began when all heifers (*n* = 15) were inoculated with Bovine Herpesvirus-1 on day 8 at h 180. ^**^P ≤ 0.05 and ^*^P > 0.05 and ≤ 0.10.

**Figure 6:**
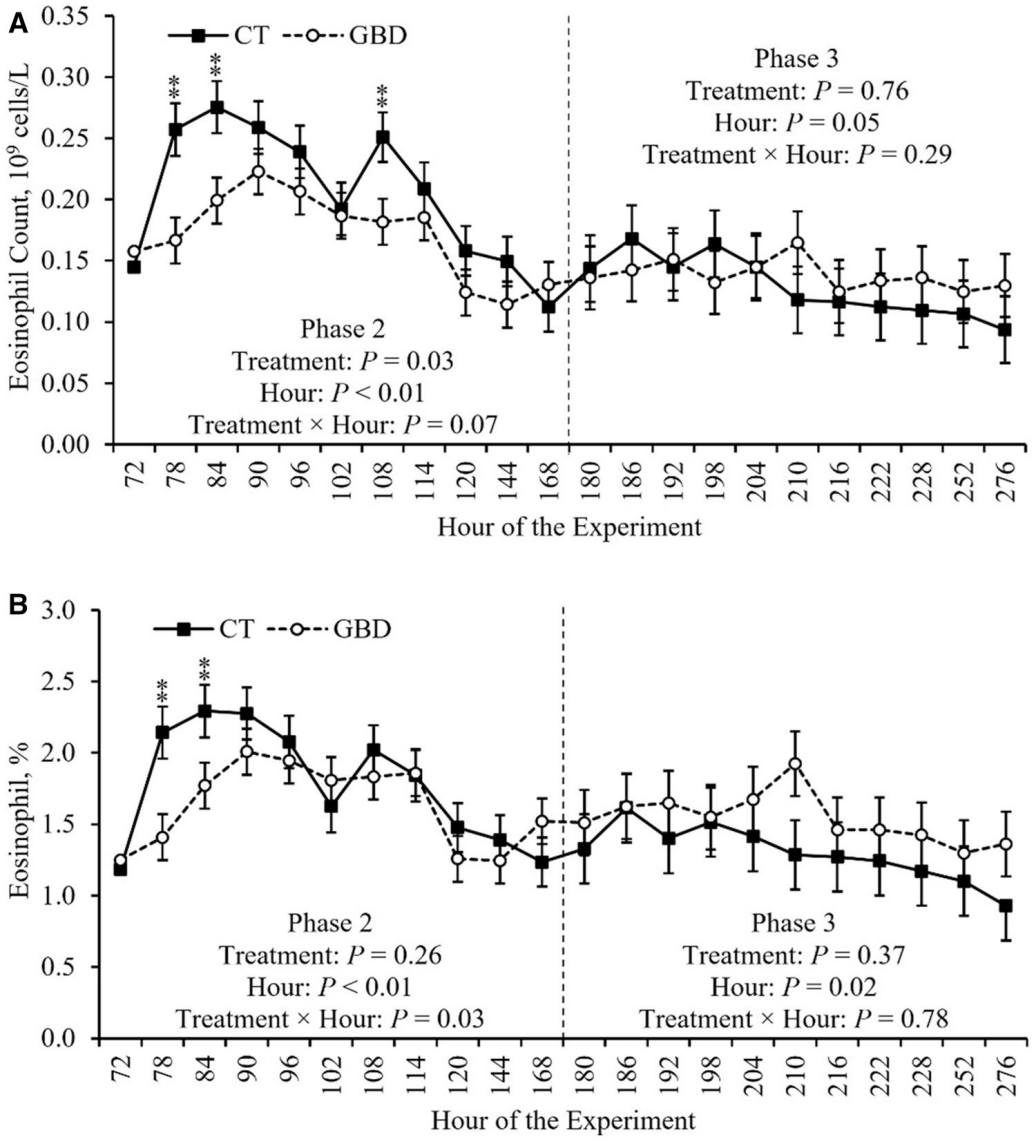
Treatment × hour effects on eosinophil count and percentage obtained by complete blood count during Phase 2. The values reported are covariate adjusted least square means. The baseline measurement immediately prior to treatment administration, hour 72, was used as a covariate for statistical analysis. Phase 2 included administering aspirin via balling gun (100 mg/kg of body weight) to the gut barrier dysfunction group (GBD; *n* = 8) every 12 h from days 4 to 7 followed by a 24 h withdrawal, and the control group (CT; *n* = 7) did not receive aspirin. Phase 3 began when all heifers (*n* = 15) were inoculated with Bovine Herpesvirus-1 on day 8 at h 180. ^**^P ≤ 0.05.

The effects of aspirin administration on CBC measurements during Phase 2 are depicted in Table 3. Treatment × hour effects of aspirin administration during Phase 2 on RBC and HGB, as well as EOS and EOS%, are depicted in Figures 5 and 6, respectively. There was a treatment × hour effect on HGB and EOS% (*P *≤ 0.03) during Phase 2 (Table 3). HGB was decreased in hours 84, 102, 108, and 168 (*P *≤ 0.05), and tended to be decreased in hour 78 (*P *= 0.08), in GBD compared to CT (Figure 5; Panel C). EOS% was decreased in hours 78 and 84 (*P *≤ 0.04) in GBD compared to CT (Figure 6; Panel B). Additionally, there was a tendency for a treatment × hour effect on EOS, RBC, and HCT (*P *≤ 0.09) during Phase 2 (Table 3). RBC was decreased (*P *= 0.02) in hour 108 in GBD compared to CT (Figure 5; Panel A). HCT was decreased in hour 108 (*P *= 0.02), and tended to be decreased in hours 78, 84, and 102 (*P *≤ 0.09), in GBD compared to CT (Figure 5; Panel B). EOS was decreased (*P *≤ 0.01) in hours 78, 84, and 108 in GBD compared to CT (Figure 6; Panel A). There were no other significant treatment × hour effects (*P *≥ 0.13) on CBC measurements during Phase 2 (Table 3).

**Table 3:** Effects of aspirin administration on heifer complete blood count measurements (Phase 2).

Item	Treatment^1^		SEM^4^	*P*-value		
	CT^2^	GBD^3^		Treatment	Hour	Treatment × Hour
**White blood cell, 10^9^ cells/L**	11.9	10.0	0.456	<0.01	<0.01	0.17
**Lymphocyte, 10^9^ cells/L**	8.64	7.45	0.278	0.01	<0.01	0.18
**Lymphocyte, %**	73.7	73.0	1.62	0.78	0.42	0.69
**Monocyte, 10^9^ cells/L**	0.247	0.297	0.04	0.38	0.48	0.13
**Monocyte, %**	2.47	2.75	0.425	0.63	0.92	0.18
**Neutrophil, 10^9^ cells/L**	2.60	2.23	0.216	0.24	0.20	0.47
**Neutrophil, %**	21.5	21.8	1.52	0.89	0.27	0.83
**Basophil, 10^9^ cells/L**	0.080	0.067	0.009	0.11	<0.01	0.17
**Basophil, %**	0.710	0.640	0.04	0.26	<0.01	0.23
**Eosinophil, 10^9^ cells/L**	0.210	0.172	0.012	0.03	<0.01	0.07
**Eosinophil, %**	1.84	1.67	0.107	0.26	<0.01	0.03
**Red blood cell, 10^12^ cells/L**	8.53	8.41	0.077	0.27	<0.01	0.09
**Hematocrit, %**	36.3	35.8	0.258	0.18	<0.01	0.09
**Red cell distribution width, fL**	32.8	32.6	0.166	0.48	0.38	0.70
**Red cell distribution width, %**	26.1	25.9	0.158	0.45	0.24	0.23
**Hemoglobin, g/dL**	10.1	9.81	0.104	0.03	<0.01	<0.01
**Mean corpuscular hemoglobin, pg/cell**	11.9	11.7	0.088	0.19	0.20	0.63
**Mean corpuscular hemoglobin concentration, g/dL**	27.7	27.4	0.357	0.16	0.32	0.70
**Mean corpuscular volume, fL**	42.9	42.7	0.09	0.08	0.03	0.75
**Platelet, 10^9^ cells/L**	303	298	5.29	0.47	0.90	0.97
**Plateletcrit, %**	0.194	0.196	0.004	0.63	0.83	0.93
**Platelet distribution width, fL**	8.35	8.98	0.442	0.14	<0.01	0.27
**Platelet distribution width, %**	30.4	31.4	0.688	0.13	<0.01	0.21
**Mean platelet volume, fL**	6.41	6.51	0.093	0.25	0.11	0.40

1 Values reported are covariate (h 72, prior to treatment administration) adjusted least square means.

2 CT (control) was not administered aspirin.

3 GBD (gut barrier dysfunction) was administered aspirin via a balling gun at a dose of 100 mg/kg of body weight every 12 h from days 4 to 7 (Phase 2).

4 Highest standard error of the mean is reported.

There was an effect of treatment during Phase 2 on WBC and LYM, in which GBD had less (*P *≤ 0.01) mean WBC and LYM compared to CT (Table 3). Additionally, there was a tendency for an effect of treatment during Phase 2 on MCV, in which GBD had decreased (*P *= 0.08) mean MCV compared to CT (Table 3). No other effects of treatment on CBC measurements were observed during Phase 2 (*P *≥ 0.11; Table 3).

There was an effect of hour on WBC, LYM, BAS, MCV, PDW, BAS%, and PDW% during Phase 2 (*P ≤ *0.03; Table 3). WBC, LYM, BAS, BAS% in both treatment groups decreased over time (*P *≤ 0.01; Table 4). Oppositely, MCV, PDW, and PDW% increased (*P *≤ 0.03) in both treatment groups over time (Table 4). There were no other effects of hour on CBC measurements observed during Phase 2 (*P *≥ 0.11; Table 3).

**Table 4:** Effects of hour on complete blood count measurements of all heifers (Phase 2).

Hour	Item^2^						
	WBC^1^	LYM^1^	BAS^1^	BAS%^1^	MCV^1^	PDW^1^	PDW%^1^
**78**	12.0^a^	8.66^a^	0.079^b^	0.648^b^	42.5^a^	8.40^a,b^	30.5^a,b^
**84**	11.9^a,b^	8.60^a,b^	0.093^a,b^	0.786^a^	42.6^a,b^	8.09^a^	30.1^a^
**90**	11.4^a,b^	8.53^a,b^	0.094^a^	0.842^a^	42.9^b^	8.51^a,b^	30.7^a,b^
**96**	11.2^b^	8.22^a,b^	0.086^a,b^	0.786^a^	43.0^b^	8.57^a,b^	30.8^a,b^
**102**	11.1^b^	8.37^a,b^	0.071^b^	0.634^b,c^	42.8^b^	8.89^b,c^	31.3^b^
**108**	11.3^b^	8.20^b^	0.083^a,b^	0.749^a,b^	42.8^b^	8.57^a,b^	30.8^a,b^
**114**	10.8^b,c^	8.00^b,c^	0.076^b^	0.750^a,b^	42.8^b^	8.85^b,c^	31.3^b^
**120**	10.5^b,c^	7.68^c^	0.054^c^	0.523^c^	42.7^a,b^	8.68^b^	31.0^b^
**144**	10.1^c^	7.23^d^	0.048^c^	0.496^c^	42.7^a,b^	8.67^b^	30.9^b^
**168**	9.42^d^	6.99^d^	0.049^c^	0.537^b,c^	43.1^b^	9.39^c^	32.0^b^

1 BAS, basophil count (10^9^ cells/L); BAS%, basophils as a percentage of white blood cells; LYM, lymphocyte count (10^9^ cells/L); MCV, mean corpuscular volume (fL); PDW, platelet distribution width (fL); PDW%, platelet distribution width ratio; WBC, white blood cell count (10^9^ cells/L).

2 Values reported are least square means.

a–d Means within each column with different superscripts are statistically different (*P *≤ 0.05).

**Figure 7:**
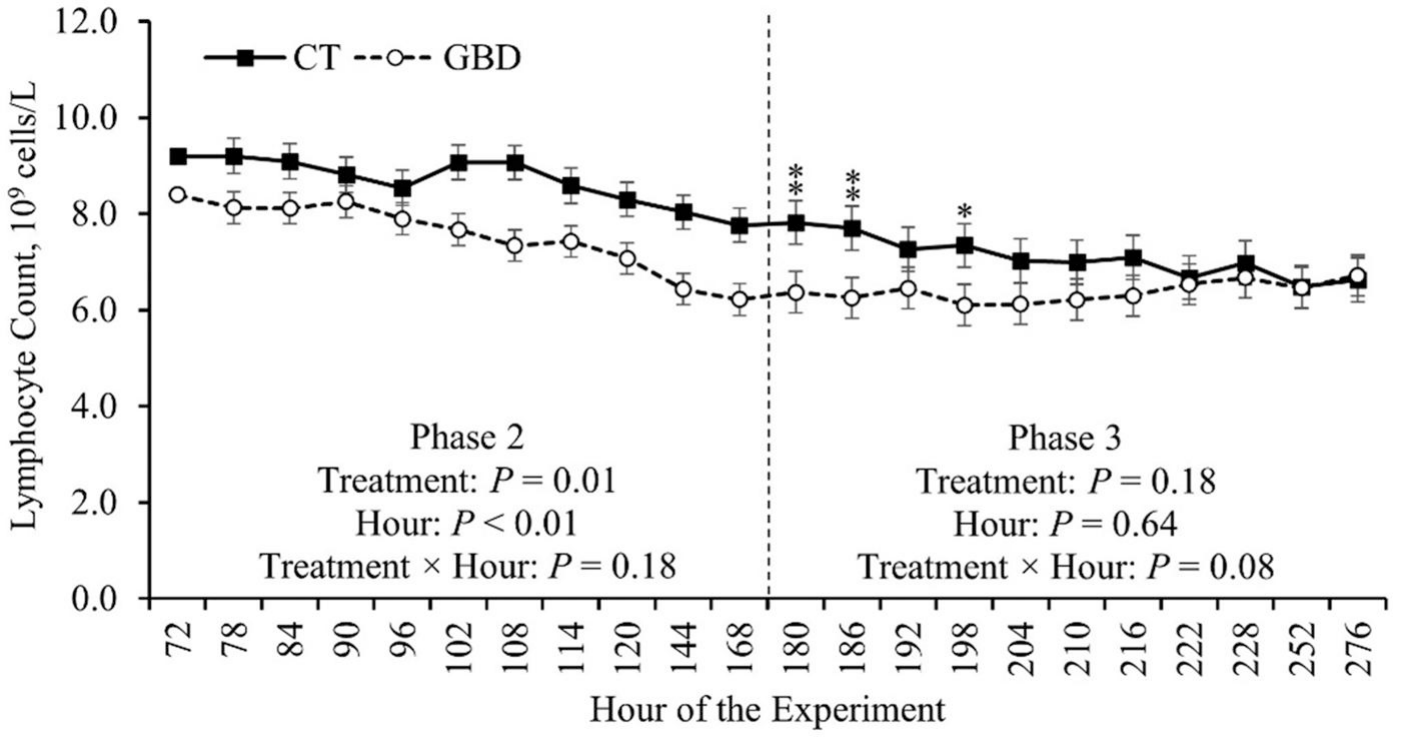
Treatment × hour effect on lymphocyte count obtained by complete blood count during Phase 3. The values reported are covariate adjusted least square means. The baseline measurement immediately prior to treatment administration, hour 72, was used as a covariate for statistical analysis. Phase 2 included administering aspirin via balling gun (100 mg/kg of body weight) to the gut barrier dysfunction group (GBD; *n* = 8) every 12 h from days 4 to 7 followed by a 24 h withdrawal, and the control group (CT; *n* = 7) did not receive aspirin. Phase 3 began when all heifers (*n* = 15) were inoculated with Bovine Herpesvirus-1 on day 8 at h 180. ^**^P ≤ 0.05 and ^*^P > 0.05 and ≤ 0.10.

The effects of the treatment on CBC measurements during the BHV-1 challenge in Phase 3 are reported in Table 5, and a tendency for a treatment × hour effect on LYM is depicted in Figure 7. There was a treatment × hour effect on LYM in Phase 3 (*P *= 0.08; Table 5). In hour 180 and 186 LYM was decreased (*P *≤ 0.03) in GBD compared to CT (Figure 7). Additionally, there was a tendency for LYM to be decreased (*P *= 0.06) in hour 198 in GBD compared to CT (Figure 7). There were no other treatment × hour effects on CBC measurements in Phase 3 (*P *≥ 0.20; Table 5).

**Table 5:** Effects of intentionally induced increased gut permeability on heifer complete blood count measurements during the respiratory disease challenge (Phase 3).

Item	Treatment^1^		SEM^4^	*P*-value		
	CT^2^	GBD^3^		Treatment	Hour	Treatment × Hour
**White blood cell, 10^9^ cells/L**	10.1	8.86	0.514	0.08	0.53	0.23
**Lymphocyte, 10^9^ cells/L**	7.09	6.38	0.351	0.18	0.64	0.08
**Lymphocyte, %**	72.0	70.2	2.14	0.55	0.14	0.83
**Monocyte, 10^9^ cells/L**	0.308	0.255	0.130	0.61	<0.01	0.82
**Monocyte, %**	3.22	2.54	1.18	0.45	<0.01	0.85
**Neutrophil, 10^9^ cells/L**	2.33	2.22	0.190	0.68	0.47	0.83
**Neutrophil, %**	22.9	25.2	2.53	0.43	0.64	0.94
**Basophil, 10^9^ cells/L**	0.053	0.061	2.31	0.61	0.25	0.37
**Basophil, %**	0.553	0.664	0.094	0.40	0.17	0.72
**Eosinophil, 10^9^ cells/L**	0.129	0.138	0.023	0.76	0.05	0.29
**Eosinophil, %**	1.30	1.54	0.190	0.37	0.02	0.78
**Red blood cell, 10^1^^2^ cells/L**	8.44	8.36	0.108	0.57	0.82	0.60
**Hematocrit, %**	36.1	35.9	0.423	0.70	0.85	0.52
**Red cell distribution width, fL**	32.6	32.5	0.472	0.88	0.22	0.54
**Red cell distribution width, %**	26.0	25.9	0.231	0.70	0.12	0.30
**Hemoglobin, g/dL**	9.88	10.1	0.257	0.62	0.50	0.65
**Mean corpuscular hemoglobin, pg/cell**	11.7	11.6	0.217	0.25	0.14	0.26
**Mean corpuscular hemoglobin concentration, g/dL**	27.3	27.0	0.351	0.11	0.05	0.20
**Mean corpuscular volume, fL**	43.1	43.1	0.249	0.79	0.92	0.21
**Platelet, 10^9^ cells/L**	329	320	20.0	0.75	0.01	0.93
**Plateletcrit, %**	0.21	0.212	0.014	0.95	<0.01	0.92
**Platelet distribution width, fL**	8.32	8.92	0.244	0.07	0.02	0.87
**Platelet distribution width, %**	30.4	31.4	0.408	0.05	0.02	0.87
**Mean platelet volume, fL**	6.42	6.54	0.055	0.14	0.03	0.48

1 Values reported are covariate (hour 72, prior to treatment administration) adjusted least square means.

2 CT (control) was not administered aspirin and was inoculated with BHV-1 at hour 180.

3 GBD (gut barrier dysfunction) was given a 24 h withdrawal from aspirin before inoculation with BHV-1 at h 180.

4 Highest standard error of the mean is reported.

There was a tendency for an effect of treatment on WBC, PDW, PDW% (*P *≤ 0.08) during Phase 3 (Table 5). GBD tended to have lower (*P *= 0.08) mean WBC compared to CT in Phase 3 (Table 5). Oppositely, GBD tended to have greater mean PDW and PDW% during Phase 3 (*P *≤ 0.07; Table 5). There were no other effects of treatment on CBC measurements during Phase 3 (*P *≥ 0.11; Table 5).

Hour effects on CBC measurements during the BHV-1 challenge in Phase 3 are reported in Table 6. There was an effect of hour on MON, MON%, EOS%, PLT, PCT, PDW, PDW%, and MPV (*P *≤ 0.03; Table 5). Overall, MON, MON%, PLT, PCT%, PDW, and PDW% increased among both treatment groups overtime (*P *≤ 0.02; Table 6). MPV increased until h 204 and then decreased thereafter (*P *= 0.03; Table 6). Oppositely, there was a decrease (*P *= 0.02) in EOS% overtime (Table 6). Additionally, there was a tendency for an effect of hour (*P *= 0.05) on EOS and MCHC (Table 5). Overall, EOS decreased (*P *= 0.05) for both treatment groups over time (Table 6). Similarly, MCHC decreased (*P *= 0.05) for both treatment groups until hour 228, but it increased thereafter (Table 6). There were no other effects of hour on CBC measurements during Phase 3 (*P *≥ 0.12; Table 5).

**Table 6:** Effects of hour on complete blood count measurements of all heifers during the respiratory disease challenge (Phase 3).

Hour^3^	Item^2^									
	MON^1^	MON%^1^	EOS^1^	EOS%^1^	MCHC^1^	PLT^1^	PCT%^1^	MPV^1^	PDW^1^	PDW%^1^
**180**	0.191^a^	2.00^a^	0.140^a,b^	1.42^a,b^	27.2^a,b^	304^a,b^	0.197^a,b^	6.41^a,b^	8.44^a^	30.6^a,b^
**186**	0.214^a^	2.27^a^	0.155^a^	1.62^a^	27.5^b^	297^a^	0.189^a^	6.35^a^	8.20^a^	30.2^a^
**192**	0.239^a^	2.32^a^	0.148^a,b^	1.53^a,b^	26.8^a^	309^a,b^	0.205^a,b^	6.60^b^	9.10^b^	31.7^b^
**198**	0.148^a^	1.59^a^	0.148^a,b^	1.53^a,b^	27.3^a,b^	336^b^	0.220^b^	6.53^b^	8.57^b^	30.9^a,b^
**204**	0.190^a^	1.95^a^	0.145^a,b^	1.54^a,b^	27.1^a,b^	333^b^	0.220^b^	6.61^b^	9.18^b^	31.7^b^
**210**	0.211^a^	2.28^a^	0.141^a,b^	1.61^a,b^	26.8^a^	334^b^	0.216^b^	6.46^a,b^	8.43^c^	30.6^a,b^
**216**	0.213^a^	2.34^a^	0.121^b^	1.37^b^	26.9^a^	329^b^	0.212^b^	6.42^a,b^	8.31^c^	30.4^a,b^
**222**	0.287^a^	2.99^a^	0.123^b^	1.35^b^	27.0^a,b^	337^b^	0.218^b^	6.47^a,b^	8.55^c^	30.8^a,b^
**228**	0.295^a^	2.93^a^	0.123^b^	1.30^b^	26.9^a^	332^b^	0.219^b^	6.53^b^	8.73^c^	31.0^a,b^
**252**	0.548^b^	5.70^b^	0.116^b^	1.20^b^	27.4^a,b^	310^a,b^	0.196^a,b^	6.35^a^	8.47^c^	30.6^a,b^
**276**	0.558^b^	5.32^b^	0.112^b^	1.15^b^	27.6^b^	351^b^	0.230^b^	6.51^a,b^	8.84^c^	31.3^b^

1 EOS, eosinophil count (10^9^ cells/L); EOS%, eosinophils as a percentage of white blood cells; MCHC, mean corpuscular hemoglobin concentration (g/dL); MON, monocyte count (10^9^ cells/L); MON%, monocytes as a percentage of white blood cells; MPV, mean platelet volume (fL); PCT, plateletcrit; PDW, platelet distribution width (fL); PDW%, platelet distribution width ratio; PLT, platelet count (10^9^ cells/L).

2 Values reported are covariate (hour 72, prior to treatment administration) adjusted least square means.

3 Samples from hour 180 were collected immediately before all heifers were inoculated with Bovine Herpesvirus-1.

a–c Values within each column with different superscripts are statistically different (*P *≤ 0.05).

### Blood biomarkers

**Figure 8:**
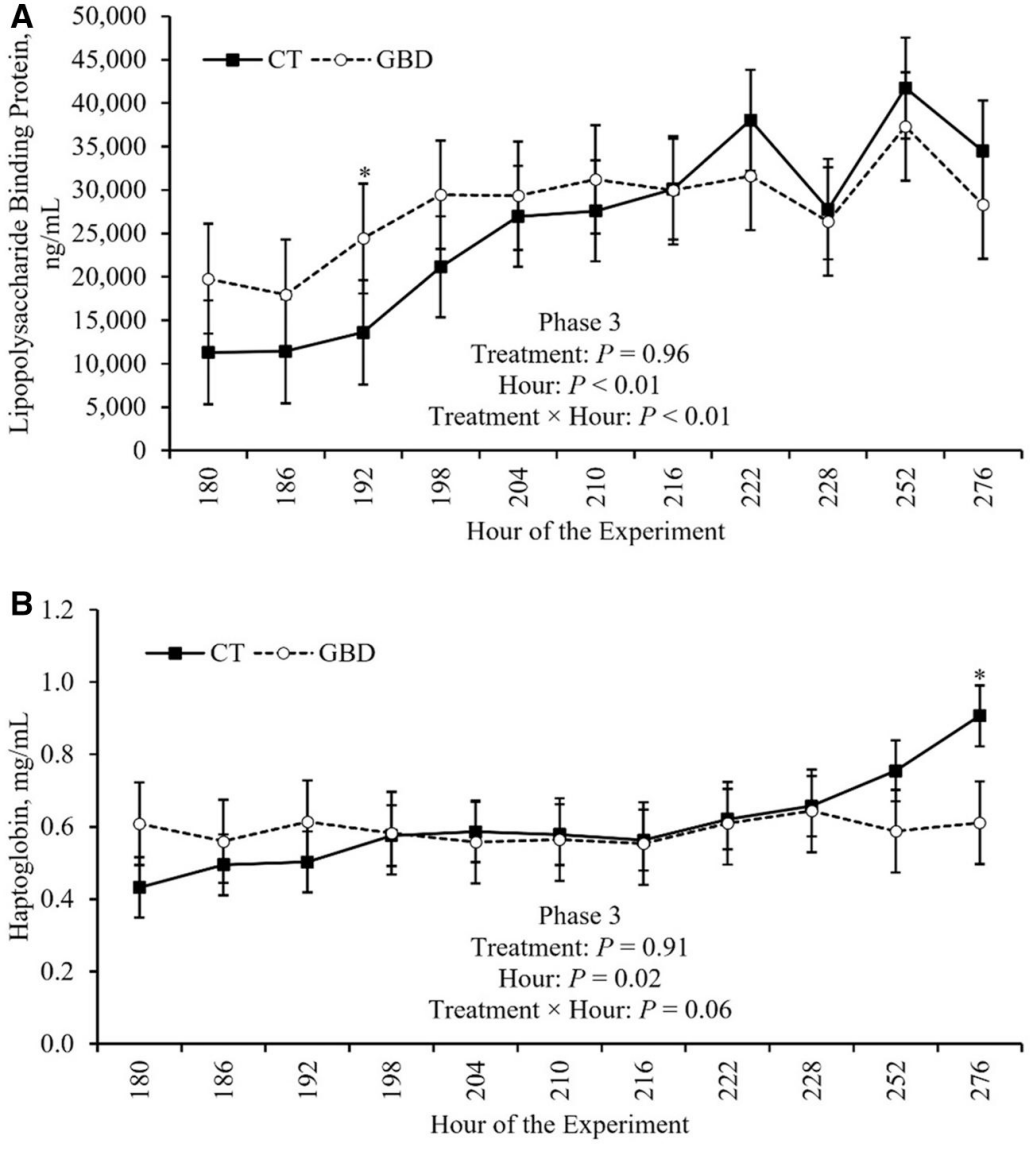
Treatment × hour effects on lipopolysaccharide-binding protein and haptoglobin concentration during Phase 3. Treatments included: GBD (*n* = 8), which were administered aspirin (100 mg/kg of BW) via balling gun every 12 h from days 4 to 7 followed by a 24 h withdrawal, and the control group (CT; *n* = 7) did not receive aspirin. Phase 3 began when all heifers (*n* = 15) were inoculated with BHV-1 on day 8 at h 180. BHV-1, bovine herpesvirus-1; BW, body weight; CT, control; GBD, gut barrier dysfunction. ^*^P > 0.05 and ≤ 0.10.

Effects of the treatment on serum and plasma concentration of biomarkers of inflammation and stress that were measured during the BHV-1 challenge in Phase 3 are reported in Table 7 and depicted in Figure 8. There was a treatment × hour effect (*P *< 0.01) on LBP concentration in Phase 3 (Table 7). In hour 192, serum LBP tended (*P *= 0.10) to be greater in GBD compared to CT (Figure 8; Panel A). Additionally, there was a tendency (*P *= 0.06) for a treatment × hour effect on plasma haptoglobin concentration (Table 7). In hour 276, plasma concentration of haptoglobin tended (*P *= 0.09) to be decreased for GBD compared to CT (Figure 8; Panel B). There were no other treatment × hour effects (*P *≥ 0.29) on concentrations of blood biomarkers of inflammation and stress measured during Phase 3 (Table 7). There was no significant effect of treatment (*P *≥ 0.19) on any of the blood biomarkers measured in Phase 3 (Table 7).

**Table 7:** Effects of intentionally induced increased gut permeability on heifer serum and plasma concentration of blood biomarkers of inflammation and stress during the respiratory disease challenge (Phase 3).

Item	Treatment^1^		SEM^4^	*P*-value		
	CT^2^	GBD^3^		Treatment	Hour	Treatment × Hour
**Interleukin-6, pg/mL**	43.8	79.9	42.1	0.54	0.09	0.58
**Interleukin-10^5^, pg/mL**	1,135	2,784	1,723	0.28	0.30	0.42
**Tumor necrosis factor-α, ng/mL**	0.995	0.044	0.677	0.32	0.40	0.90
**Lipopolysaccharide binding protein^5^, ng/mL**	25,836	27,805	5,719	0.96	<0.01	<0.01
**Haptoglobin, mg/mL**	0.607	0.590	0.104	0.91	0.15	0.06
**Serum amyloid A, μg/mL**	221,133	235,578	24,791	0.68	<0.01	0.29
**Cortisol, ng/mL**	11.0	8.78	1.23	0.20	0.37	0.49

1 Values reported are least square means.

2 CT (control) was not administered aspirin and was inoculated with BHV-1 at h 180.

3 GBD (gut barrier dysfunction) was given a 24 h withdrawal from aspirin before inoculation with BHV-1 at h 180.

4 Highest standard error of the mean is reported.

5 Log transformation was used for statistical analysis.

Hour effects on concentrations of blood biomarkers that were measured during the BHV-1 challenge in Phase 3 are depicted in Table 8. There was an effect of hour (*P *< 0.01) on serum SAA concentration during Phase 3. Serum SAA concentration increased in the hours following the BHV-1 challenge before peaking at hour 198 and decreasing dramatically to its minimum concentration by hour 210, where it then increased gradually throughout the remainder of Phase 3 (Table 8). Additionally, there was a tendency (*P *= 0.09) for an effect of hour on serum IL-6 concentration (Table 7). Serum IL-6 concentration neared its peak at hour 204 and decreased dramatically to its minimum concentration by hour 210, where it then increased gradually for the remainder of Phase 3, achieving its maximum concentration at hour 276 (Table 8).

**Table 8:** Effects of hour on serum concentrations of interleukin-6 and serum amyloid A of all heifers during the respiratory disease challenge (Phase 3).

Hour^3^	Item^2^	
	IL-6^1^	SAA^1^
**180**	84.4^a^	203,983^b^
**186**	60.0^a,b^	210,608^b^
**192**	51.7^a,b^	306,093^c,d^
**198**	65.4^a,b^	371,240^d^
**204**	88.8^a^	298,400^c^
**210**	13.1^b^	128,863^a^
**216**	15.4^b^	142,682^a^
**222**	35.9^a,b^	158,697^a,b^
**228**	87.3^a^	232,141^b^
**252**	87.7^a^	230,975^b^
**276**	90.4^a^	228,226^b^

1 IL-6, interleukin-6 (pg/mL); SAA, serum amyloid A (μg/mL).

2 Values reported are least square means.

3 Samples from h 180 were collected immediately before all heifers were inoculated with BHV-1.

a–d Values within each column with different superscripts are statistically different (*P *≤ 0.05).

### Metrics of the acute-phase response

All heifers exhibited some shared characteristics indicative of an acute-phase response during BHV-1 challenge in Phase 3, which are depicted in Figure 10 (descriptive information). Though there was not a significant effect of hour on TNF-α (*P *= 0.40) or cortisol (*P *= 0.37) concentration (Table 7), concentrations of TNF-α, IL-6, SAA, and cortisol all peaked, or approached their peak, between 18 and 24 h (hours 198 and 204) after BHV-1 inoculation (Figure 10; Panels A, B, and D, respectively). Conversely, while the effect of hour was not significant for IL-10 (*P *= 0.30), its concentration peaked 36 hours (hour 216) after BHV-1 inoculation, as TNF-α, IL-6, and SAA decreased dramatically (Figure 10; Panels A and B). LBP and haptoglobin increased more gradually over Phase 3 and approached their peaks toward the end of the experiment (Figure 10; Panel B).

During Phase 3, all heifers experienced a dramatic increase in MON (effect of hour; *P *< 0.01; Table 5) of nearly 3-fold from hour 180 to 276, but NEU (effect of hour; *P *= 0.47; Table 5) responses were unremarkable (Figure 10; Panel C). Additionally, decreasing DMI accompanied increasing DVT throughout Phase 3 (Table 2).

### qPCR

The qPCR results for BHV-1 in Phase 3 are reported in Table 9. There were no treatment or treatment × hour effects (*P *≥ 0.28) on Ct values in Phase 3 (Table 9). There was an effect of hour (*P *> 0.01) on qPCR results, in which Ct value increased for both treatments following BHV-1 inoculation at hour 180 (Table 9).

**Table 9:** Heifer cycle threshold (Ct) values of qPCR for BHV-1 during the respiratory disease challenge (Phase 3).

Item	Treatment^1^, Ct Value			SEM^4^	*P*-value		
	Average	CT^2^	GBD^3^		Treatment	Hour	Treatment × Hour
**h 180^4^**	0^a^	0	0	0.884	–	–	–
**h 228**	23.93^b^	24.8	23.1	0.886	–	–	–
**h 288**	24.74^b^	26.1	23.4	0.886	–	–	–
**Phase 3**	–	–	–	–	0.28	<0.001	0.40

1 Values reported are least square means.

2 CT (control) was not administered aspirin and was inoculated with BHV-1 at h 180.

3 GBD (gut barrier dysfunction) was given a 24 h withdrawal from aspirin before inoculation with BHV-1 at h 180.

4 Samples from h 180 were collected immediately before all heifers were inoculated with BHV-1.

a,b Values within each column with different superscripts are statistically different (*P *≤ 0.05).

### Intestinal morphology

**Figure 9:**
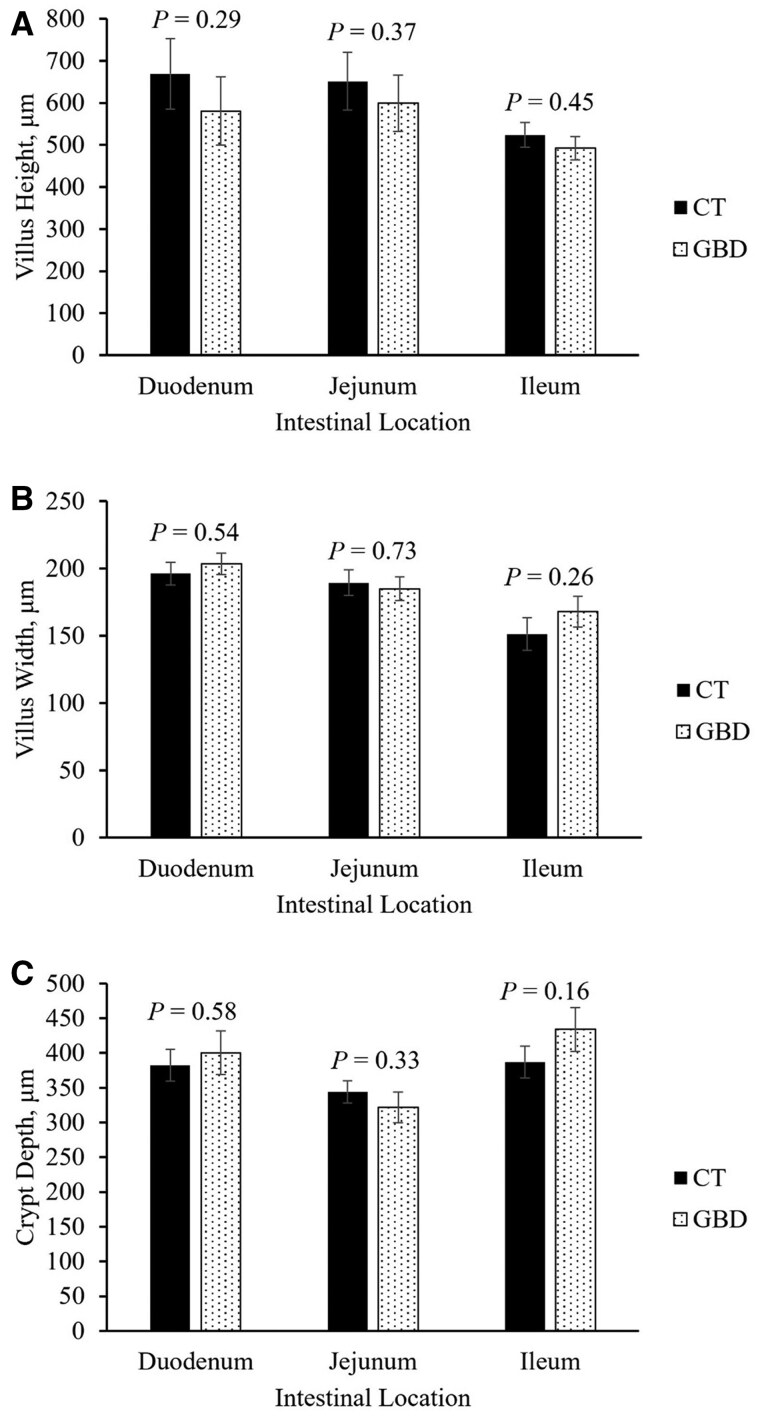
Effect of intentionally induced increased gastrointestinal permeability on small intestine morphology by intestinal location. Treatments included GBD (*n* = 8), which were administered aspirin (100 mg/kg of BW) via balling gun every 12 h from days 4 to 7 followed by a 24 h withdrawal; and the control group (CT; *n* = 7) did not receive aspirin. All heifers (*n* = 15) were euthanized at the end of the experiment to collect tissues from the duodenum, jejunum, and ileum of the small intestine. Samples were processed, hematoxylin and eosin stained, and digitized to compare morphometric measurements of villus height, villus depth, and crypt depth at each intestinal location. Ten measurements were taken per slide of the intestinal region, resulting in 30 total measurements per heifer. BW, body weight; CT, control; GBD, gut barrier dysfunction.

The effects of the treatment on intestinal morphology at the end of the experiment are depicted in Figure 9. There was no effect of the treatment on villus height (*P *≥ 0.29; Panel A), villus width (*P *≥ 0.26; Panel B), or crypt depth (*P *≥ 0.16; Panel C) in the duodenum, jejunum, or ileum (Figure 9). However, the villus height: crypt depth ratio in the ileum was lower in GBD compared with CT (1.23 vs. 1.63; *P* = 0.03; data not shown).

**Figure 10:**
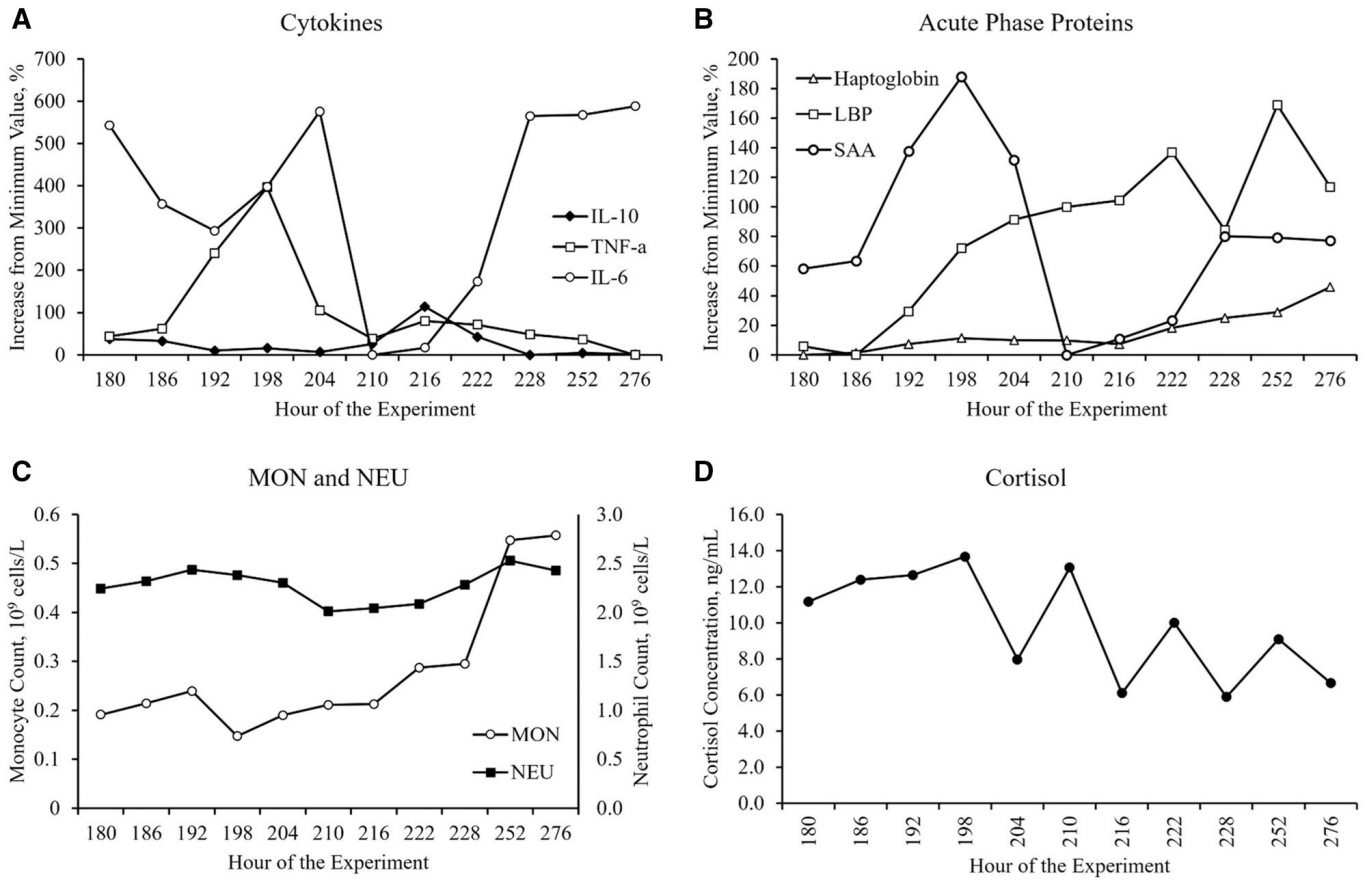
Metrics depicting the acute-phase response of all heifers during Phase 3. Values reported are the least square means average of both treatment groups. All heifers (*n* = 15) were inoculated with Bovine Herpesvirus-1 on day 8 at h 180. CT, control; GBD, gut barrier dysfunction; IL-6, interleukin-6; IL-10, interleukin-10; LBP, lipopolysaccharide binding protein; MON; monocyte count; NEU, neutrophil count; SAA, serum amyloid A; TNF-α, tumor necrosis factor alpha.

### Intestinal gene expression

The relative mRNA expression of target genes in the ileum of the heifers used this study is presented (back-transformed from the log2 used in the statistical analysis) in Table 10. The biological function of the target genes measured in this study is depicted in Table S3. Inflammatory-related genes measured, including *FOXP3*, *IL10*, *NFKB*, *TNFA*, *TLR1*, *TLR2*, *TLR3*, and *TLR4*, did not differ between groups (*P *≥ 0.12). Similarly, no statistical differences were observed in relative mRNA expression of intestinal epithelia markers *CLDN1*, *CLDN4*, *TPJ1*, and *OCLN1* (*P* ≥ 0.17).

**Table 10:** Effects of intentionally induced increased gut permeability on the relative mRNA expression of inflammatory and intestinal integrity-related genes in the ileum of heifers during the respiratory disease challenge (Phase 3).

Gene symbol^1^	Treatment^2^		SEM^5^	*P*-value
	CT^3^	GBD^4^		
**Inflammatory response**				
***FOXP3***	0.05	0.10	3.076	0.31
***IL10***	1.57	2.56	0.675	0.23
***NFKB***	1.23	1.80	1.709	0.24
***TNFA***	1.95	3.87	1.192	0.30
***TRL1***	1.50	2.76	0.818	0.12
***TLR2***	1.39	3.79	1.556	0.22
***TLR3***	0.59	0.95	1.760	0.29
***TLR4***	2.11	4.14	1.256	0.21
**Intestinal epithelial markers**				
***FABP2***	0.98	1.83	1.694	0.36
***CLDN1***	2.34	4.58	1.658	0.20
***CLDN4***	0.93	2.52	0.811	0.17
***TPJ1***	1.66	3.35	1.078	0.27
***OCLN***	1.12	2.51	0.841	0.30

1 Gene names and their respective biological functions are depicted in Table S3.

2 Values reported are the means relative mRNA expression (back-transformed; log transformation was used for statistical analysis).

3 CT (control) was not administered aspirin and was inoculated with BHV-1 at h 180.

4 GBD (gut barrier dysfunction) was given a 24 h withdrawal from aspirin before inoculation with BHV-1 at h 180.

5 Highest standard error of the mean is reported.

## Discussion

### The use of NSAIDs in GBD models

Nonsteroidal anti-inflammatory drugs (NSAIDs), particularly aspirin, has been used in research models to induce GBD due to their well-documented gastrointestinal side effects (Bjarnason et al. 1986; Briggs et al. 2020; Briggs et al. 2021; Cangiano et al. 2022). Aspirin is an NSAID commonly used for its analgesic, anti-inflammatory, and antipyretic properties, which are achieved through a pharmacological action involving competitive active site inhibition of cyclooxygenase (COX) enzymes. COX-1 and -2 are enzymes that have an important role as mediators of homeostasis and inflammation through the conversion of arachidonic acid to prostaglandin H2, a prostaglandin intermediate, and its subsequent conversion to prostanoids (Bancos et al. 2009; Ricciotti and Fitzgerald 2011). A commonly recognized side effect of COX non-selective NSAID usage is gastrointestinal toxicity (Cryer and Kimmey 1998; Radi and Khan 2006; Bancos et al. 2009), and detrimental symptoms such as gastric ulceration, erosions, bleeding, and perforations have been associated with NSAID usage in humans and animals (Radi and Khan 2006).

In cattle production, aspirin was previously directed for use at a maximum recommended dosage of 100 mg/kg of BW every 12 h (Gingerich et al. 1975) and required a 24 h meat and milk withdrawal time (Smith et al. 2008). It is important to note that despite the availability of aspirin products ostensibly for livestock use, recently (2024), the FDA released a press notice confirming there are no approved aspirin products for use in cattle, and the extra-label use of any unapproved drugs in food-producing animals is prohibited. However, aspirin remains an effective tool in research settings to induce and study GBD. When dosed to feedlot cattle at rates of up to 200 mg/kg of BW per day, aspirin caused increased production of SAA and LBP, and increased expression of claudin-1 in the small intestine (Briggs et al. 2020). These results can be achieved as soon as 72 h after aspirin administration, after one single dose of 100 mg/kg of aspirin, and are indicative of increased permeation of inflammatory endotoxins and increased turnover of tight junction proteins (Briggs et al. 2020). In the same study, administration of 100 mg/kg of aspirin at 12 h intervals within one single day was the optimal dosage proposed for increasing gut permeability as measured by the recovery of Cr-EDTA, dosed 48 h after the last dose of aspirin, in the urine (Briggs et al. 2020). The dosage rate of 100 mg/kg of aspirin every 12 h for four days was accordingly adopted for this experiment. Notably, the effects of aspirin on the gut barrier are sustained for a considerable period of time and have been observed for up to 16 months after discontinuation (Bjarnason et al. 1987; Briggs et al. 2020).

### Observations during phase 2—aspirin administration

In Phase 2 of this experiment, aspirin was administrated at a therapeutically effective dosage (100 mg/kg of BW; Gingerich et al. 1975) for 4 d to evaluate its impact on cattle performance and health. At this dosage rate and duration, aspirin administration caused a slight reduction of feed intake toward the latter end of Phase 2. However, another study using aspirin to induce GBD in feedlot cattle over a 159-d feeding period, reported no effect on feed intake over a longer period aspirin administration (Briggs et al. 2021). It is worth noting that Briggs et al. (2021) mixed aspirin into the total mixed ration rather than delivery through an oral bolus, and the dosage used by these authors based on feed intake was about half (50.7 mg/kg of BW) of the dosage used in our experiment (100 mg/kg of BW). Therefore, the cause of this observation is not immediately clear, but our results finding that aspirin decreased DMI with increasing days of administration would seem to conflict with prior studies and will require further investigation to affirm.

Further effects of aspirin administration were observed during Phase 2 on hemogram measurements. The effects of aspirin on red blood cell production and morphology has been reported previously, in which NSAID exposure decreased the diameter and surface area to volume ratio of red blood cells by disrupting their phospholipid bilayer (Bergaglio et al. 2023). Additionally, NSAID usage has been linked to decreased hemoglobin levels, which is used as a diagnostic indicator of gastrointestinal toxicity (Goldstein et al. 2011). In this way, NSAID usage may impose further consequences on tissues of the gastrointestinal tract and throughout the body by affecting the ability of red blood cells to efficiently transport oxygen (Bergaglio et al. 2023).

The DVT increased for all heifers prior to the viral challenge because this period involved several procedures known to elevate body temperature, including placement of jugular catheters, relocation into an enclosed barn, and frequent handling for intensive blood sampling. Aspirin administration also exhibited effects between treatments in terms of reducing DVT and altering the leukogram, which is consistent with the indication of aspirin as an anti-pyretic and anti-inflammatory drug across many species (Radi and Khan 2006). Pertaining to these results, NSAIDs are often used as an adjunct therapy with antibiotics in the treatment of BRD to reduce pyrexia and abnormal clinical signs (Francoz et al. 2012; De Koster et al. 2022). Fever reduction can be achieved by NSAIDs largely through inhibition of prostaglandin E_2_, which plays an important role in the cardinal features of inflammation, such as swelling, pain, redness, and fever (Bancos et al. 2009; Ricciotti and Fitzgerald 2011). While in the interest of improving animal welfare, this strategy may be beneficial in the short term, some potential immunological consequences of NSAID administration on the resolution of diseases have been proposed. Though hyperinflammatory responses can be problematic in their own right, the basis of the argument against NSAID usage during active infection with disease asserts that reducing the immune response which the body utilizes to combat pathogens may impair efficient disease resolution (Apley 2006; Bancos et al. 2009), and fever reduction can be detrimental towards disease outcomes (Carroll and Forsberg 2007; Horst et al. 2021). We are inclined to concur with these concerns as revealed through the observed leukogram alterations during aspirin administration that appeared to be driven in large part by reductions in average LYM and decreases in EOS seen in the beginning and middle of Phase 2. This effect is likely caused by alterations in cytokine production, which are crucial for leukocyte activation and recruitment, caused by COX inhibition and subsequent prostaglandin suppression (Bancos et al. 2009). Notably, prostaglandin D_2,_ which is released by mast cells, can mediate the recruitment of lymphocytes and eosinophils directly via the prostaglandin D_2_ receptor 2 expressed on T helper type 2 cells (Ricciotti and Fitzgerald 2011), and thus may have been a key factor in this observation. If the decrease in LYM also involves decreases in B-cell count, it may correspond with previous literature that links the use of NSAIDs with decreased antibody production in cattle and humans (Opelz et al. 1973; Bancos et al. 2009; Sockett et al. 2013; Chen et al. 2021). For example, ibuprofen, another NSAID, has been observed to reduce the production of human IgM and IgG antibodies by 97% and 70% in vitro, respectively, which is thought to be caused by COX-2-mediated alterations in B-cell activation (Bancos et al. 2009). Additionally, it has been reported that calves treated for BRD with a combination of antibiotic and NSAID drugs had lower serum IgG compared to those that were not treated for BRD, which was not the case for the group treated for BRD with an antibiotic alone (Sockett et al. 2013). For these reasons, judicious use of NSAIDs during active disease states should be considered to account for the potentially negative impacts on immune functions.

Independent of aspirin administration, both treatment groups exhibited some similar trends in CBC measurements during Phase 2, for which there are no definitive explanations. Increases in BAS and EOS early in Phase 2, were followed by a sustained decrease over time thereafter. In a similar manner, both treatments exhibited decreasing WBC and LYM over time during Phase 2. While cortisol levels were not measured during Phase 2, simultaneous decreases in EOS and LYM are some characteristics of a “stress leukogram” caused by increased concentration of endogenous glucocorticoids (Roland et al. 2014). This may be indicative of some degree of stress response brought about by surgical catheterization and handling in the early days of the experiment.

### Indications of GBD following aspirin administration

Following the end of aspirin administration in Phase 2, total tract gastrointestinal permeability was compared using Cr-EDTA, an indigestible marker, as a metric of paracellular permeability. In this experiment, we chose to analyze the recovery of Cr-EDTA via blood samples, rather than urine, to avoid the potential for catheter-associated urinary tract infections that can result from long-term indwelling urinary catheterization (Werneburg 2022). The concentration of Cr-EDTA recovered in plasma samples was considerably lower than in other similar experiments that analyzed plasma or serum Cr-EDTA concentrations (Amado et al. 2019; Horst et al. 2020; Cangiano et al. 2022; Pisoni et al. 2022). In previous studies, Cr‑EDTA dosing was based on a volumetric approach, and in the current experiment, the administered amount was calculated using the gravimetric weight of the solution. This resulted in a lower dosage rate of Cr-EDTA being administered to each heifer compared to the other studies. Still, the heifers that received aspirin had increased recovery of Cr-EDTA as determined by the area under the curve of peripheral blood samples over time. Recovery of Cr-EDTA from both blood and urine have been used to determine total tract gastrointestinal permeability, and this outcome concurs with previous experiments using aspirin to increase gastrointestinal permeability (Briggs et al. 2020; Briggs et al. 2021), and another experiment that increased the difference of Cr-EDTA concentration recovered in serum before and after the administration of indomethacin (Cangiano et al. 2022), another NSAID. Our results suggest that aspirin administration altered gastrointestinal permeability in our treatment group sufficiently to increase permeability to Cr-EDTA and presumably immunogenic pathogen-associated molecular patterns such as lipopolysaccharide (LPS) and lipoteichoic acid. LPS is a component of the cell wall of gram-negative bacteria, that is bound in the bloodstream by LBP which facilitates subsequent associations with cluster of differentiation 14 (i.e. CD-14) and the toll-like receptor 4 (i.e. TLR4)/myeloid differentiation factor-2 complex of immune cells to induce signals that elicit innate immune activation (Lu et al. 2008; Ceciliani et al. 2012). LPS is the pathogen-associated molecular pattern that is most often implicated in driving inflammation due to increased epithelial permeability (Mani et al. 2012). However, it is important to note that while increased recovery of Cr-EDTA may indicate some degree of increased epithelial permeability, it is not an ideal metric of increased LPS translocation alone (Briggs et al. 2021). This is because Cr-EDTA has a lesser mass of 340 Da compared to LPS; which is approximately 10 to 20 kDa for monomers and up to >1,000 kDa for micelles (García-Lafuente et al. 2001; Mani et al. 2012; Briggs et al. 2020; Hannon and Prina‐Mello 2021; Silva et al. 2023). However, the assumptions made about increased permeability to bacterial LPS in this experiment are supported by the concomitant tendency for an increase in plasma LBP concentration that is seen at the beginning of Phase 3 among the GBD group. LBP is often used as a by-proxy indicator of increased bacterial endotoxin presence in the place of LPS itself due to the challenges associated with LPS assays (Bischoff et al. 2014; La Torre et al. 2023). Because of the role of LBP in the LPS-driven inflammation cascade, increasing LBP concentration is linked to increased levels of bacterial LPS in blood circulation (Khafipour et al. 2009).

### Observations during phase 3—viral respiratory disease challenge

Phase 3 of the experiment began with the inoculation of all heifers at hour 180 with the BHV-1 virus, after allowing the treatment group a 24-h withdrawal period from aspirin as recommended by Smith et al. (2008) for meat and milk withdrawal. Aspirin derived salicylates have a rapid half-life of 32 min in cattle (Gingerich et al. 1975), thus this withdrawal period was speculated to be sufficient to minimize the carryover effects as an anti-inflammatory drug. In contrast, the detrimental effects of aspirin on the gastrointestinal epithelial barrier are expected to persist for a much greater time after withdrawal, as they have been observed for up to 16 months after discontinuation of aspirin usage (Bjarnason et al. 1987; Briggs et al. 2020). Thus, Phase 3 allowed us to examine the effects of increased gastrointestinal permeability on DMI, DVT, immunological parameters (e.g. CBC, cytokines, acute-phase proteins, and cortisol), viral shedding (e.g. qPCR), and alterations to the villi of the small intestine caused by the treatment to induce GBD during the disease challenge.

In the days following inoculation with BHV-1, all heifers exhibited signs of an effective respiratory disease challenge. Both treatment groups tested positive on qPCR for BHV-1 via nasal swabs that contained abundant amounts of the target nucleic acid (indicated by a Ct value between 1.0 and 28.0). Both groups exhibited visible signs of morbidity associated with BHV-1, in which the heifers appeared depressed (data not shown), vaginal temperature increased (0.8°C increase on average from Phase 2 to Phase 3), feed intake was dramatically decreased (36.7% decrease on average from Phase 2 to Phase 3), and lesions became visible in the nasal mucosa (data not shown). These observations were further supported through immunological and CBC observations that included increases in pro-inflammatory cytokine concentrations, acute-phase protein concentrations, and MON.

The term “acute-phase response” describes the onset of the body’s early-defenses orchestrated by the innate immune system which may be triggered by a variety of stimuli including disease, stress, trauma, infection, neoplasia, and inflammation (Carroll and Forsberg 2007; Cray et al. 2009). All heifers exhibited some shared characteristics of a typical acute-phase response in cattle during Phase 3. Namely, they experienced increases in body temperature, monocyte count, pro-inflammatory cytokine concentration, and acute-phase protein production (Carroll et al. 2009). Early in Phase 3, TNF-α, IL-6, and SAA all peaked at or near the same time. While the effect of hour was not significant for TNF-α, the numerical increase of IL-6 and TNF-α concentration was as much as 588% and 397%, respectively, from their minimum concentration. TNF-α and IL-6 are pro-inflammatory cytokines that are commonly cited as indicators of the acute-phase response, and their concentrations are known to change rapidly (Sharon et al. 2019). SAA is a major acute-phase protein of the acute-phase response, and its concentration usually begins to increase within 4 hours of pathogen recognition and often peaks within 24–48 h (Trela et al. 2022). Conversely, down regulation of the acute-phase response appeared to occur around hour 210 with a spike in IL-10 concentration, which is an anti-inflammatory cytokine that dampens the cell mediated response (Murphy and Weaver 2016). When TNF-α, IL-6, SAA, and IL-10 are used as the primary parameters for deciphering the acute-phase immune response, this process was observed to have occurred within the first 36 h after inoculation (hours 180 to 216). Additionally, while often less remarked on, cortisol has a role in the acute-phase response (Smock et al. 2023). Transient increases in cortisol coincide with increases in pro-inflammatory cytokines at the onset of inflammatory response (Cooke 2017; Smock et al. 2023). However, sustained increases in cortisol concentration exert potent anti-inflammatory effects and classically function as a part of the resolution of inflammatory response (Cooke 2017; Wilson et al. 2017). Cortisol appeared to slightly increase during the acute-phase response, peaking at hour 198 simultaneously with TNF-α concentration. Transient increases in cortisol concentration during the acute-phase response in this experiment are much more subtle in comparison to others, which have reported increases in cortisol concentration as much as 294% (Smock et al. 2023) to 3,127% (Carroll et al. 2009) from the baseline during an LPS challenge. Still, the average cortisol concentration for both treatment groups exceeded the reference value for cattle of 5–10 ng/mL (Villa et al. 2021) at their peak in the hours following inoculation.

After the initial acute-phase immune response, innate immune activation was characterized by a gradual increase in IL-6 concentration, a major promoter of the febrile response, acute-phase protein production, and monocyte activation (Eckersall et al. 1999; Murphy and Weaver 2016). Increasing from the trough in hour 210, IL-6 appeared to elicit the increases in DVT, MON, SAA, LBP, and haptoglobin concentration that occurred at the latter end of Phase 3. LBP increased gradually over time during Phase 3 for all heifers and peak values for both groups occurred toward the latter end of the sampling period at hour 252. Thus, they may not have reached their maximum potential within our experimental period. Haptoglobin levels remained relatively constant for the GBD group, but increased over time for the CT group, with the peak for CT occurring at the last timepoint, which suggests again that this increasing trend for the CT group may not have reached its maximum potential within our experimental period. MON increased nearly 3-fold from hour 180 to 270 on average for both treatment groups. Interestingly, neither group exhibited any remarkable changes in NEU during our experiment. Along with monocytes, neutrophils are often characterized by their crucial role in the innate immune response (Liew and Kubes 2019; Smock et al. 2023). Some have described BHV-1 infections as having inhibitory effects on the activity of phagocytic cells such as monocytes and neutrophils (Leite et al. 2004), but the considerable increase in MON would seem to contradict this theory. Instead, neutrophils are often described as playing a more vital role in bacterial, fungal, and protozoal infections (Liew and Kubes 2019), rather than viral infections, and thus may have had a limited role in the early stages of a viral disease challenge.

Both treatment groups exhibited increases in acute-phase protein concentrations in Phase 3. In their respective roles, these acute-phase proteins can provide insights into the immune response during the disease challenge. Increases in acute-phase protein production is stimulated by the pro-inflammatory cytokines: TNF-α, interleukin-1β, and especially IL-6 (Murphy and Weaver 2016; Saco and Bassols 2023). Acute-phase proteins are generally classified into three categories: major (10 to 100 fold increase within 48 h followed by rapid decline), moderate (2 to 10 fold increase with prolonged duration), or minor (slight increase with prolonged duration), and they display functional diversity across species with distinct patterns and roles in the immune response (Eckersall et al. 1999; Trela et al. 2022). All three acute-phase proteins examined in this experiment are often classified as major acute-phase protein in cattle (Saco and Bassols 2023)—though some have classified LBP as moderate (Ceciliani et al. 2012)—yet their production throughout the acute-phase response often involves some discrepancies (Eckersall et al. 1999; Cray et al. 2009), which appear to manifest among our results. SAA concentration peaked much earlier, as a part of the initial acute-phase response, compared to haptoglobin. This pattern is consistent with previous literature that proports increases in SAA are induced earlier on in an infection compared to haptoglobin (Eckersall et al. 1999), which is better used as an indicator of inflammation over time (Sharon et al. 2019). LBP concentration increased gradually over time, but similar to haptoglobin, peaked much later in Phase 3. SAA and haptoglobin are the most frequently cited major positive acute-phase proteins in cattle, and they act as key indicators of infection (Eckersall et al. 1999; Cray et al. 2009; Gifford et al. 2012). The role of SAA in the immune response is often characterized by, but not limited to, its role in modulating the innate immune response, scavenging cholesterol from dying cells, and opsonizing target pathogens and cells to promote their elimination by phagocytes (Ceciliani et al. 2012). Haptoglobin functions especially to scavenge free hemoglobin and nitric oxide in the blood to limit oxidative damage and reduce the availability of iron that may otherwise be used for bacterial growth (Ceciliani et al. 2012). LBP has a key role in immunomodulation by facilitating the recognition and binding of bacterial LPS and lipoteichoic acid and activating innate immune system activities (Briggs et al. 2020) and serves as a crucial component in the body’s ability to recognize and resolve bacterial infection (Ceciliani et al. 2012).

Some CBC measurement trends during Phase 3 that were shared by the treatment groups are less conclusive. Both treatment groups exhibited decreasing EOS and EOS% over time. While changes in EOS are most often associated with parasitic infection and allergen induced hypersensitivity reactions, eosinopenia can result from increased concentrations of endogenous glucocorticoids (Roland et al. 2014), as mentioned previously. Though, in this instance, cortisol concentration decreased over time after acute-phase response downregulation. However, eosinopenia has also been reported to occur early in episodes of acute inflammation, including viral infections, which may be a more apt explanation for this observation (Bass et al. 1980; Roland et al. 2014). It is unclear whether the increases in PLT, PCT, and platelet indices such as PDW, PDW%, and MPV over the course of Phase 3 among all heifers were related to inflammation (Roland et al. 2014), are an artifact from the process of sample collection, or if they appear as a result of local thrombophlebitis induced by the prolonged presence of an intravenous catheter. MCHC also tended to increase for all heifers, but the cause of this observation is not well understood.

### Consequences of increased gut permeability on the immune response

The combination of increased Cr-EDTA recovery and increased LBP concentration early in Phase 3 serves as indicators of an effective GBD challenge induced by aspirin administration (Briggs et al. 2021), and suggest that there was an increased baseline LPS load in the GBD going into Phase 3. We observed that the GBD group exhibited greater LBP concentration at hour 192, and LBP concentration was numerically ≥57% greater in early Phase 3 from hours 180 to 192 compared to the CT group. On this basis, we can examine how gut derived inflammation due to GBD affects the immune response throughout Phase 3. It is important to note, however, that LPS can enter circulation through other epithelial barriers than the gut alone, such as the respiratory tract (Khafipour et al. 2009), and influence LBP concentrations.

Respiratory infections with viral pathogens (such as BHV-1) often increase susceptibility to bacterial pneumonia in cattle and contribute to the pathogenesis of the BRD complex (Leite et al. 2004; Gaudino et al. 2022). Toward the latter portion of Phase 3, increases in LBP concentration occurred for both treatment groups over time, presumably driven by increased LPS pressure from a secondary opportunistic bacterial infection. However, only the CT group exhibited increased haptoglobin concentration during Phase 3. Because haptoglobin is a key acute-phase protein in the bovine immune response (Eckersall et al. 1999; Gifford et al. 2012), the lower haptoglobin concentrations observed in the GBD group compared to controls could hinder the efficiency of disease resolution. While further research will be necessary to elucidate the reason for the lack of increase, we speculate that continuous inflammation over time from increased gut permeability elevates energy expenditure considerably, and strains the limited resources needed for activation of the immune response, which requires significant resources to maintain (Mani et al. 2012; Cooke 2017; Kvidera et al. 2017b). The effects of GBD on energy expenditure would be especially pronounced, because the gut is the body’s largest immune organ (Mani et al. 2012; Stewart et al. 2017), and LPS derived inflammation can increase energy expenditure by 30% and lead to negative nitrogen balances (Lochmiller and Deerenberg 2000; Mani et al. 2012). The negative effects of GBD on the immune system are presumably even greater during a simultaneous episode of respiratory disease when feed and water intake is depressed as a result of acute inflammation (Carroll and Forsberg 2007; Mani et al. 2012; Cooke 2017), thereby further straining nutrient availability. An alternative explanation is that GBD heifers may have mounted an earlier innate immune response following BHV‑1 challenge, leading to adequate haptoglobin production earlier in the inflammatory process and reducing the need for further hepatic APP synthesis during the later sampling period.

Another potential example of the consequences of increased energy expenditure derived from GBD exists in our observation that the GBD group tended to have decreased average DVT compared to CT across the BHV-1 challenge. While both treatment groups experienced elevated core body temperature (greater than 39.5°C) over time following the BHV-1 challenge, only the CT group experienced what many consider to be a clinical fever in cattle (indicated by a core body temperature greater than 40°C). The GBD group never reached this temperature threshold in Phase 3. While the possibility of carry-over effects from aspirin administration during Phase 2 cannot be removed entirely, this outcome may suggest that the ability to mount a febrile response to the BHV-1 challenge was suppressed among the GBD group. This observation is not entirely surprising if our energy expenditure hypothesis is correct, because the febrile response is a very energetically demanding process; for example, the increase in basal energy expenditure needed to raise the core body temperature by 1°C has been reported to be as much as 13% (Kluger 1978; Mani et al. 2012). In practice, this observation could also have implications when making the decision whether to administer treatment, as in many cases a rectal temperature threshold of ≥40°C is used when diagnosing BRD and determining whether antibiotic therapy is warranted (Edwards 2010; Blakebrough-Hall et al. 2020; De Koster et al. 2022; Galyean et al. 2022). Additionally, the febrile response plays an important role in the immune response by destroying and/or reducing the growth of some bacteria and microorganisms as well as accelerating immune cell proliferation and enzymatic processes (Carroll and Forsberg 2007). For these reasons, suppressed febrile responses can result in increased rates of negative health outcomes among diseased animals (Carroll and Forsberg 2007; Horst et al. 2021).

When comparing qPCR results, there were no statistical differences between treatment groups, but some trends are worth noting. qPCR values are used as a measurement of pathogen load (Tom and Mina 2020; Penney et al. 2022), and the cycle threshold (Ct) value is inversely proportional to the amount of the target nucleic acid present in the sample. Thus, a lower Ct value is indicative of a greater amount of the target nucleic acid, and presumably a greater amount of the pathogen, in the sample. All heifers tested negative (indicated by a Ct value of zero) for BHV-1 prior to inoculation (hour 180), and positive in the middle (hour 228) and end (hour 288) of Phase 3. On a numerical basis, the Ct value of the CT group increased more between hours 228 and 288 than the GBD group, which showed relatively little change. If the experiment was able to be conducted for a longer period, seeing if this trend continued over time could yield a consequential observation.

At the end of this experiment, our treatment did not appear to exhibit any significant effects on morphometric measurements of the villi in the small intestine; contrary to the results of other experiments pertaining to GBD (Kvidera et al. 2017a; Cangiano et al. 2022). For example, Cangiano et al. (2022) reported that indomethacin injections reduced villus length and surface area in the small intestine and reduced the depth and width of villus crypts in the colon. However, our observation of increased Cr-EDTA recovery and LBP concentration among the GBD group indicates that there was a considerable effect of aspirin administration on overall gut permeability. With that in mind, changes to villus morphology having developed over a longer period of aspirin administration remain a possibility. It should also be noted that alterations to gut permeability and morphology can also occur outside of the small intestine, such as the rumen or the colon (Emmanuel et al. 2007; Zhang et al. 2013; Cangiano et al. 2022), which were not examined in this experiment. Within this scenario, although the inflammatory and intestinal integrity gene markers in the ileum tissue did not statistically differ between groups, their substantial numerical differences are worth noting. In our study, the numerically higher ileal expression of *CLDN1* and *CLDN4* in GBD heifers can be attributed to increased gut permeability. As shown in human and rodent model studies, increased expression of *CLDN1* during inflammatory bowel disease and *CLDN4* in certain cancer types are associated with increased inflammatory activity and increased intestinal barrier dysfunction (Michl et al. 2003; Weber et al. 2008; Amasheh et al. 2009).

### Comparing results

In other experiments that involved NSAID administration, some have reported no effect (Briggs et al. 2020; Briggs et al. 2021), or an increase in IL-6 concentration among the increased gut permeability treatment groups (Cangiano et al. 2022). IL-6 concentration increased for both treatments during the disease challenge, but we could not report an effect of GBD on IL-6 concentration in this experiment. Similarly, Cangiano et al. (2022) reported an increase in IL-10 concentration in the blood of calves due to indomethacin administration, while in this study, *IL10* (an anti-inflammatory cytokine) had numerically higher expression in the ileum of GBD heifers, which can be partially attributed to the anti-inflammatory properties of NSAID administration.

Whether using an NSAID or a gamma secretase inhibitor to induce GBD, increased LBP concentration among cattle with increased gut permeability is frequently reported (Kvidera et al. 2017a; Briggs et al. 2020; Briggs et al. 2021), which is consistent with our results. However, this difference was only seen early in Phase 3, which we interpreted as an indication of increased baseline inflammation. Over time, the CT group achieved similar, and even slightly numerically increased LBP concentration over the course of the disease challenge. When comparing SAA results, others reported either increased SAA concentration in the increased gut permeability group (Briggs et al. 2020; Cangiano et al. 2022), or no difference between treatments (Kvidera et al. 2017a; Briggs et al. 2021). In our experiment, both groups experienced increased SAA concentration during the immune challenge, but no difference was found between treatments, and thus we cannot affirm any effect of GBD on SAA concentration. Notably, others have reported no effect of increased gut permeability on haptoglobin concentration (Kvidera et al. 2017a; Briggs et al. 2020; Briggs et al. 2021). To our knowledge, the tendency for GBD to cause inhibitory effects on haptoglobin production that was observed in our experiment has not been otherwise reported. This development among our insights is likely due to the novel introduction of the disease challenge. Furthermore, others have also reported either an increase in their metric of core body temperature due to increased gut permeability (Kvidera et al. 2017a), or no effect (Cangiano et al. 2022), which is contrary to our results. During the disease challenge, the GBD group tended to exhibit a decreased ability to mount a similar febrile response to the CT group. To our knowledge, this is also the first time an effect of GBD on decreasing the febrile response has been reported. Again, this is likely due to this being the first GBD experiment with cattle, that we are aware of, that has included a disease challenge.

### Limitations

Hopefully, some known limitations of this experiment, listed hereafter, can be addressed in future studies. We also acknowledge that a BHV-1 challenge represents only the viral component of the broader BRD complex. The sample size used in this study, based on Cr recovery from Briggs et al. (2021) for intestinal permeability, may have prevented, to some extent, the achievement of statistical significance between groups for certain parameters. Additionally, due to the limited timeframe in which cattle can be subjected to catheterization without adverse consequences, this experiment was not able to study the immune response to the greatest potential extent. Most of the immune responses that were observed during the disease challenge are presumably derived primarily from the innate, or non-specific, immune system. Initial indicators of the adaptive immune response generally do not appear for 4–7 d after the onset of disease and therefore could not be sufficiently explored in this experiment (Janeway Jr et al. 2001). If the design of the experiment would have been able to permit a more prolonged sampling period, exploring the ways in which adaptive immune responses are impacted by increased gut permeability would provide worthwhile insight. Further, while the NSAID model has been used numerous times to study GBD (Bjarnason et al. 1986; Briggs et al. 2020; Briggs et al. 2021; Cangiano et al. 2022), one cannot be certain as to the congruency of this model to the pathogenesis of episodes of GBD derived from nature, even if they generally result in similar outcomes (Cangiano et al. 2022). Lastly, the influence of stress that is inherent to these kinds of experiments imposes a challenge in producing experimental groups that serve as a proper negative control, as stress is a factor in the etiology of GBD (Söderholm and Perdue 2001; Pohl et al. 2017; Horst et al. 2021). As technology improves, the ability to conduct such studies with minimal animal handling may help to limit the confounding influence of stress in GBD research in the future.

## Conclusions

We have demonstrated that administering aspirin to cattle can increase gut permeability as indicated by an increase in Cr-EDTA recovery and LBP production. Furthermore, we report that increased gut permeability may influence the innate immune response when cattle are challenged with respiratory disease, most notably by suppressing the febrile response and altering acute-phase protein production. In addition to the performance-related consequences of increased gut inflammation, which are better understood, our results suggest that the influence of gut health on the immune response may affect outcomes when cattle are faced with disease. Future studies should examine how increased gut permeability affects energy metabolism and immune responses to further elucidate its impact on disease outcomes during incidents of BRD. These findings suggest that greater consideration is owed to gut health in our efforts to reduce the impact of BRD on the cattle industry.

## Supplementary Material

skag117_Supplementary_Data

## Abbreviations

APP: acute-phase protein
BAS: basophil count
BAS%: basophils as a percentage of white blood cells
BHV-1: bovine herpesvirus-1
BRD: bovine respiratory disease
BW: body weight
CBC: complete blood count
CIDR: controlled intravaginal drug release
COX: Cyclooxygenase
Cr-EDTA: chromium-ethylenediaminetetraacetic acid
Ct: cycle threshold
DMI: dry matter intake
DVT: average daily vaginal temperature
ELISA: enzyme-linked immunosorbent assay
EOS: eosinophil count
EOS%: eosinophils as a percentage of white blood cells
HCT%: hematocrit
GBD: gut barrier dysfunction
IL-6: interleukin-6
IL-10: interleukin-10
LBP: lipopolysaccharide binding protein
LPS: lipopolysaccharide
LYM: lymphocyte count
LYM%: lymphocytes as a percentage of white blood cells
MCH: mean corpuscular hemoglobin
MCHC: mean corpuscular hemoglobin concentration
MCV: mean corpuscular volume
MON: monocyte count
MON%: monocytes as a percentage of white blood cells
MPV: mean platelet volume
NEU: neutrophil count
NEU%: neutrophils as a percentage of white blood cells
NSAID: nonsteroidal anti-inflammatory drug
PDW: platelet distribution width
PDW%: platelet distribution width ratio
PCT%: plateletcrit
PLT: platelet count
qPCR: quantitative polymerase chain reaction
RBC: red blood cell count
RDW: red cell distribution width
RDW%: red cell distribution width ratio
SAA: serum amyloid A
TNF-α: tumor necrosis factor- α
WBC: white blood cell count
