# Supplementary material for: Effects of gut barrier dysfunction during a viral respiratory disease challenge on immune function of feedlot beef calves
Source: J Anim Sci. 2026 Apr 10;104:skag117. doi: 10.1093/jas/skag117 (PMC13152583; doi:10.1093/jas/skag117)
Supplement: skag117_Supplementary_Data [file skag117_supplementary_data.zip › Foster_2025_Supplemental Table 1.docx]

**Supplemental Table 1**. Detailed information on the total RNA isolated from the Ileum tissue of the heifers on experimental day 13

| RNA (ng/µL)^1^ | A260/280^2^ | RINe score^3^ |
| --- | --- | --- |
| 348.17 | 2.10 | 5.8 |
| 465.24 | 2.14 | 6.2 |
| 962.50 | 2.08 | 4.6 |
| 617.61 | 2.09 | NA |
| 575.05 | 2.07 | 6.1 |
| 681.80 | 2.08 | 6.6 |
| 804.91 | 2.09 | 7.1 |
| 344.24 | 2.09 | NA |
| 620.51 | 2.11 | 5.5 |
| 504.77 | 2.15 | 5.9 |
| 718.00 | 2.07 | 5.5 |
| 678.56 | 2.07 | 3.0 |
| 567.34 | 2.19 | 6.9 |
| 648.18 | 2.07 | NA |
| 1186.10 | 2.12 | 7.5 |

^1^ total RNA from was eluted in a final volume of 50 µL.

^2^ A260/A280 ratio is a widely used indicator of nucleic acid purity in spectrophotometry. A ratio of ~2.0 is considered pure.

^3^ RINe = RNA Integrity Number equivalent; RINe is a score obtained from the RNA ScreenTape assay (TapeStation System, Agilent Technologies) that serves as a key quality control metric, indicating the degree of RNA degradation.
