# Supplementary material for: Effects of gut barrier dysfunction during a viral respiratory disease challenge on immune function of feedlot beef calves
Source: J Anim Sci. 2026 Apr 10;104:skag117. doi: 10.1093/jas/skag117 (PMC13152583; doi:10.1093/jas/skag117)
Supplement: skag117_Supplementary_Data [file skag117_supplementary_data.zip › Foster_2025_Supplemental Table 3.docx]

**Supplemental Table 3**. Detailed information on the selected internal control genes and target genes measured in this study. Gene symbols and names, and gene biological functions were obtained from the National Center for Biotechnology Information Database (NCBI)

| Gene Symbol | Gene Name | Biological function and process |
| --- | --- | --- |
| *ACTB* | actin beta | Highly conserved protein that is involved in cell motility, structure, integrity, and intercellular signaling. |
| *B2M* | beta-2-microglobulin | B2M encodes a serum protein found in association with the major histocompatibility complex (MHC) class I heavy chain on the surface of nearly all nucleated cells. |
| *TLR1* | toll-like receptor 1 | TLRs recognize pathogen-associated molecular patterns (PAMPs) that are expressed on infectious agents and mediate the production of cytokines necessary for the development of effective immunity. The various TLRs exhibit different patterns of expression. |
| *TLR2* | toll-like receptor 2 | TLR2 is a cell-surface protein that can form heterodimers with other TLR family members to recognize PAMPs and modulate the host's inflammatory response. |
| *TLR3* | toll-like receptor 3 | TLR3 recognizes dsRNA and PAMPS and induces the activation of NFKB and the production of type I interferons. |
| *TLR4* | toll-like receptor 4 | TLR4 recognizes pathogen-associated molecular patterns that are expressed on infectious agents. TLR4 has been found to have a high binding affinity for lipopolysaccharide (LPS) found in gram-negative bacteria. |
| *FOXP3* | forkhead box P3 | The protein encoded by FOXP3 is a crucial transcription factor for regulatory T cells. |
| *TNFA* | tumor necrosis factor | TNFA encodes a multifunctional proinflammatory cytokine secreted by macrophages that is involved in the regulation of a wide spectrum of biological processes, including cell proliferation, differentiation, apoptosis, lipid metabolism, and coagulation. |
| *NFKB* | nuclear factor kappa B subunit 1 | NFKB is a transcription regulator that is activated by various intra- and extracellular stimuli such as cytokines and bacterial or viral products. |
| *IL10* | interleukin 10 | IL10 encodes a cytokine produced primarily by monocytes and to a lesser extent by lymphocytes. This cytokine has pleiotropic effects in immunoregulation and inflammation. |
| *FABP2* | fatty acid binding protein 2 | The protein encoded by FABP2 is an intracellular fatty acid-binding protein that participates in the uptake, intracellular metabolism, transport of long-chain fatty acids, and in the modulation of cell growth and proliferation. |
| *CLDN1* | claudin 1 | The protein encoded by CLDN1 is an integral membrane protein and a component of tight junction strands. Tight junctions form continuous seals around cells. |
| *CLDN4* | claudin 4 | CLDN4 encodes integral membrane proteins that are components of the epithelial cell tight junctions, which regulate the movement of solutes and ions through the paracellular space. |
| *TJP1* | tight junction protein 1 | TJP1 encodes a tight junction adaptor protein and regulates adherens junctions to regulate the movement of ions and macromolecules. |
| *OCLN* | occludin | The protein encoded by OCLN is an integral membrane protein that is required for cytokine-induced regulation of the tight junction paracellular permeability barrier. |
