# Supplementary material for: Effects of gut barrier dysfunction during a viral respiratory disease challenge on immune function of feedlot beef calves
Source: J Anim Sci. 2026 Apr 10;104:skag117. doi: 10.1093/jas/skag117 (PMC13152583; doi:10.1093/jas/skag117)
Supplement: skag117_Supplementary_Data [file skag117_supplementary_data.zip › Foster_2025_Supplemental Table 2.docx]

**Supplemental Table 2.** Gene symbol and sequence of primers (5’-3’) for *Bos Taurus* used to analyze gene expression in the ileum tissue

| Gene Symbol | Forward primer (5’-3’) | Reverse primer (5’-3’) | Source |
| --- | --- | --- | --- |
| *ACTB* | CAAGGACCTGCTACGCCAACA | ACATCTGCTGGAAGGTGGAC | Kadegowda et al. (2009) |
| *B2M* | TCCAGCGTCCTCCAAAGATT | CCCATACACATAGCAGTTCAGGTAA | Kadegowda et al. (2009) |
| *TLR1* | CTGCCCATATGCCAAGAGTT | GGCATCTTCTCTTTCCCCAT | Charavaryamath et al. (2011) |
| *TLR2* | CTGTGTGCGTCTTCCTCAGA | TCAGGGAGCAGAGTAACCAGA | Charavaryamath et al. (2011) |
| *TLR3* | TCTTTTCGGGACTGTTGACC | AAATCCCCCCATCCAAGGTAG | Charavaryamath et al. (2011) |
| *TLR4* | GGTTTCCACAAAAGCCGTAA | AGGACGATGAAGATGATGCC | Charavaryamath et al. (2011) |
| *FOXP3* | CACAACCTGAGCCTGCACAA | TCTTGCGGAACTCAAACTCATC | Liang et al. (2016) |
| *TNFA* | GCCCCCAGGGCTCCAGAAGT | AGCGTGGTGGCTCCTGCAAC | Liang et al. (2016) |
| *NFKB* | AAGAGAAGATGGGGAAAGGCTG | CGTCGGCAAATGAGAAGTAGTG | Jacometo et al. (2015) |
| *IL10* | GAAGGACCAAACTGCACAGCTT | AAAACTGGATCATTTCCGACAAG | Liang et al. (2016) |
| *FABP2* | GTGGCGAGATGGTCCAGACT | TCTGTGTTCTGGGCAATGCTC | Rosa et al. (2021) |
| *TJP1* | GCACATAGGATCCCTGAACCA | TGCTTCCGGTAGTACTCCTCATC | Minuti et al. (2015) |
| *CLDN1* | GGCATCCTGCTGGGACTAATAG | CAGCCATCCGCATCTTCTGT | Minuti et al. (2015) |
| *CLDN4* | CCCCAGCCAGCAACTACGT | TCACAGATTGCAGTGAGCTCAGT | Minuti et al. (2015) |
| *OCLN* | ACGCAGGAAGTGCCTTTGGTAGC | GCAGCCATGGCCAGCAGGAA | Malmuthuge et al. (2013) |
